# Inflammatory macrophage-derived itaconate inhibits DNA demethylase TET2 to prevent excessive osteoclast activation in rheumatoid arthritis

**DOI:** 10.1038/s41413-025-00437-w

**Published:** 2025-06-11

**Authors:** Kewei Rong, Dezheng Wang, Xiting Pu, Cheng Zhang, Pu Zhang, Xiankun Cao, Jinglin Zheng, Xiao Yang, Kexin Liu, Lei Shi, Yin Li, Peixiang Ma, Dan Ye, Jie Zhao, Pu Wang, An Qin

**Affiliations:** 1 Department of Orthopedics, Shanghai Key Laboratory of Orthopedics Implant, the Ninth People’s Hospital, Shanghai Jiao Tong University School of Medicine, Shanghai, China; 2 Huashan Hospital, Fudan University, and Shanghai Key Laboratory of Medical Epigenetics, International Co-laboratory of Medical Epigenetics and Metabolism (Ministry of Science and Technology), and Molecular and Cell Biology Laboratory, Institutes of Biomedical Sciences, Shanghai Medical College of Fudan University, Shanghai, China; 3 Division of Gastroenterology and Hepatology, Key Laboratory of Gastroenterology and Hepatology, Ministry of Health, State Key Laboratory for Oncogenes and Related Genes, Renji Hospital, School of Medicine, Shanghai Jiao Tong University, Shanghai, China; 4 Shanghai Institute of Digestive Disease, Shanghai, China

**Keywords:** Bone, Pathogenesis

## Abstract

Itaconate, a macrophage-specific anti-inflammatory metabolite, has recently emerged as a critical regulator in rheumatoid arthritis pathogenesis. We found that itaconate is a TNF-α responsive metabolite significantly elevated in the serum and synovial fluid of rheumatoid arthritis patients and we demonstrated that itaconate is primarily produced by inflammatory macrophages rather than osteoclasts or osteoblasts. In TNF-transgenic and *Irg1*^*−/−*^ hybrid mice, a more severe bone destruction phenotype was observed. Administration of itaconate prevents excessive activation of osteoclasts by inhibiting Tet2 enzyme activity. Furthermore, exogenous administration of itaconate or its derivative, 4-octyl-itaconate, inhibits arthritis progression and mitigates bone destruction, offering a potential therapeutic strategy for rheumatoid arthritis. This study elucidates that TNF-α drives macrophage-derived itaconate production to epigenetically suppress osteoclast hyperactivation through Tet2 inhibition, establishing itaconate and its derivative OI as novel therapeutic agents against rheumatoid arthritis -associated bone destruction.

## Introduction

Rheumatoid arthritis (RA) is a systemic autoimmune disorder primarily characterized by chronic inflammation of the joints, which progressively leads to bone destruction and joint deformities, resulting in multiple articular disabilities.^1^ A key factor in RA pathogenesis is the activation of inflammatory macrophages, which secrete a range of pro-inflammatory cytokines, such as tumor necrosis factor-alpha (TNF-α). This cytokine plays a pivotal role in mediating both articular cartilage damage and systemic bone loss.^2^ To counteract these destructive processes, TNF-α neutralizing therapies, such as Adalimumab and Infliximab, have been developed in clinical practice to treat RA-associated inflammation and joint erosion.^3^

During RA development, metabolic dysregulation is characterized by elevated energy demands and altered oxygen and nutrient consumption within damaged tissues, especially under chronic inflammatory conditions.^4^ This metabolic shift is particularly prominent in the synovial lining, where synovial tissue macrophages and fibroblast-like synoviocytes contribute to immune cell infiltration and release high levels of pro-inflammatory cytokines and tissue-degrading enzymes. These processes intensify the destruction of cartilage and bone, creating profound changes in the local metabolic environment.^5,6^ Metabolic reprogramming is indispensable for orchestrating the activation of immune cells and bone cells in RA.^7,8^ Metabolites that are altered in RA, such as lactate, succinate, and L-arginine, have been shown to play important roles in modulating the bone tissue microenvironment by influencing inflammation, immune responses, and tissue remodeling.^9–11^ Therefore, elucidating the specific metabolic pathways involved in RA and their impact on disease progression is significant for developing novel and effective therapies for RA patients.

During the pathogenesis of RA, various cells and their secreted cytokines play significant roles. Fibroblast-like synoviocytes, cells of the innate immune system (including monocytes, macrophages, mast cells, dendritic cells, etc.), and cells of the adaptive immune system (such as T cells and B cells), as well as the cytokines they secrete, interact to drive the cascade of inflammation in the synovium. Current research on abnormal inflammatory mediators, such as interleukins (ILs), has important implications for restoring immune homeostasis in RA.^12,13^ Monocyte/macrophage infiltration of the synovium has been identified as a central feature of RA’s inflammatory pathophysiology.^14^ Local microenvironmental signals are crucial in macrophage activation, promoting functional polarization toward the classical M1 or alternative M2 phenotypes. The imbalance between pro-inflammatory M1 macrophages and anti-inflammatory M2 macrophages is associated with the exacerbation of RA. The increased secretion of pro-inflammatory cytokines by M1 macrophages contributes to the intensification of RA flares, while the release of anti-inflammatory cytokines by M2 macrophages appears to reverse the inflammatory state. Promoting the M1-M2 shift in macrophages represents a novel strategy to block RA progression and characterizes the remission phase of RA.^15–17^

Itaconate (ITA) is a metabolite produced by the mitochondrial enzyme cis-aconitate decarboxylase (ACOD1, also known as Irg1), which is highly expressed in macrophages under inflammatory conditions. ITA has emerged as a crucial regulator of immune responses and cellular metabolism,^18,19^ and its regulatory functions are mediated through multiple mechanisms (as recently reviewed^19,20^). Structurally, ITA is similar to α-ketoglutarate (α-KG) and thus can competitively bind to and inhibit α-KG-dependent epigenetic enzymes, such as DNA demethylase TET2,^21^, thereby influencing gene expression. In addition, ITA has been reported to modify proteins by covalently conjugating to cysteine residues, which can alter the function of many proteins involved in cellular signaling and immune regulation, such as KEAP1, JAK1, and NLRP3.^22–24^

Recently, ITA was reported to participate in the development of RA, but its precise function and underlying mechanisms remain unclear. One study reported that osteoclast-derived ITA inhibited osteoclast differentiation in a cell-autonomous manner, evidenced by *Irg1*-deficient mice exhibiting an increased number of osteoclasts and more severe bone erosion in experimental arthritis models.^25^ Conversely, another study reported that osteoclast-derived ITA did not directly influence osteoclast differentiation but was instead secreted extracellularly and influenced osteoblast differentiation,^26^ and that *Irg1*-deficient mice showed no difference in osteoclast numbers but experienced reduced new bone formation at sites of inflammation. This inconsistency highlights unresolved questions regarding the cell types responsible for ITA production, its target cells, and the underlying molecular mechanisms in RA. In this study, we provide both ex vivo and in vivo evidence that macrophages residing in bone tissues are the primary producers of ITA during RA development. ITA is secreted into the microenvironment, where it inhibits osteoclast activity by modulating Tet2-mediated DNA demethylation and gene expression. Finally, we show that ITA supplementation ameliorates RA symptoms, suggesting that ITA holds promise as a therapeutic metabolite for further investigation in the treatment of RA.

## Results

### Itaconate is a signature metabolite of rheumatoid arthritis

**Fig. 1:**
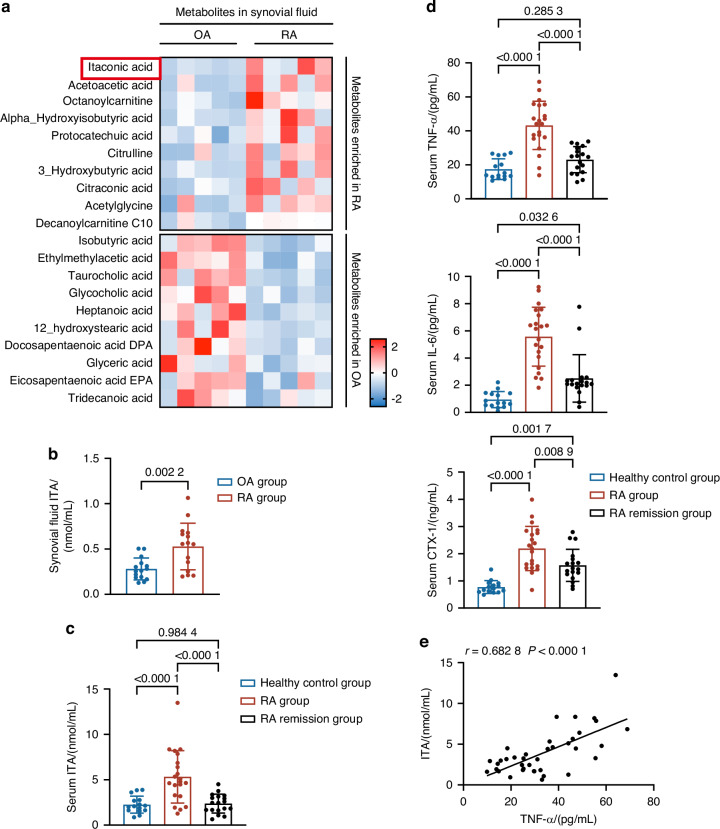
The levels of ITA are elevated in the serum and synovial fluid of patients with RA. **a** Q300 Metabolomic heatmap of synovial fluid in OA and RA patients (*n* = 5). **b** Quantitative detection of synovial fluid ITA in OA and RA patients (*n* = 15). **c**, **d** Quantitative detection of ITA (**c**), TNF-α, IL-6, and CTX-1 (**d**) in the serum of the healthy control group (*n* = 15), the RA active group (*n* = 21) and the RA remission group (*n* = 18). **e** Correlation analysis between serum ITA levels and serum TNF-α levels in the RA active group and the RA remission group (*n* = 39). Significant differences were determined by Student’s *t*-test (**a**, **b**), one-way ANOVA (**c**, **d**), and Pearson correlation (**e**). Data represent means ± SD for each group

To characterize the metabolic signature of RA, we conducted LC/MS-based metabolome analysis on synovial fluid from patients with RA and osteoarthritis (OA), and found that ITA emerged as one of the most significantly increased metabolites in RA patients (Figs. 1a and S1a). The increased ITA was further confirmed in another cohort of 15 paired OA and RA patients’ samples (Fig. 1b). In addition, we also measured ITA levels in the serum of RA patients across different disease stages. Our data demonstrated that ITA levels were notably elevated in newly diagnosed, untreated RA patients (RA active group) compared with healthy controls and patients in remission after methotrexate (MTX) treatment (Fig. 1c). In parallel, inflammatory cytokines, such as TNF-α and IL-6, as well as the bone metabolism marker C-terminal telopeptide of type I collagen (CTX-1), were found to be elevated in the serum of RA patients (Fig. 1d). Correlation analysis revealed that serum ITA levels were positively associated with various inflammatory markers, including C-reactive protein (CRP), Erythrocyte Sedimentation Rate (ESR), Disease Activity Score 28 (DAS28)-CRP, IL-6, and TNF-α, with the strongest correlation observed between ITA and TNF-α (Figs. 1e and S1b).

To further explore the role of Irg1/ITA in RA, we re-analyzed a single-cell RNA sequencing (scRNA-seq) dataset from Synapse, which included 314 011 cells from 70 RA patients and 9 OA patients.^27^ Transcriptome analysis revealed that the proportion of *IRG1*-expressing (IRG1^+^) myeloid cells was significantly elevated in RA patients compared with OA patients (Fig. S1c, e). *IRG1* expression was found to be specifically enriched in a subgroup of myeloid cells characterized by *STAT1*^*+*^*CXCL10*^*+*^ marker genes (Fig. S1d). In this subtype, 4.76% of myeloid cells were *IRG1*^*+*^ in RA patients, compared with only 0.46% in OA patients (Fig. S1f). Kyoto Encyclopedia of Genes and Genomes (KEGG) analysis of *IRG1*^*high*^ versus *IRG1*^*low*^ macrophages identified the TNF signaling pathway as the most enriched pathway in *IRG1*^*high*^ cells (Fig. S1g), suggesting a potential link between TNF-α regulation, IRG1 expression, and ITA production in the context of RA.

### *Irg1* deficiency induces a more severe bone destruction phenotype in the TNF-Tg-induced RA mouse model

Supporting the notion that *Irg1* is responsible for ITA production in macrophages during inflammatory conditions, we observed robust induction of Irg1 expression in mouse bone marrow-derived macrophages (BMMs) following stimulation with lipopolysaccharide (LPS). Notably, TNF-α, a key inflammatory cytokine in RA, also significantly induced *Irg1* expression in macrophages, indicating that TNF-α is one of the triggers for Irg1 activation in RA (Fig. S1h, i). These results are in line with our findings in RA patient samples and support the close link between TNF-α regulation and Irg1/ITA induction in inflammatory macrophages.

To assess whether *Irg1* deficiency affects normal bone development, we performed micro-computed tomography (μCT) analysis of tibiae from 12-week-old male mice, which showed no significant differences in bone structure between *Irg1*^*+/+*^ and *Irg1*^*−/−*^ mice (Fig. S2a, b). Von Kossa staining, tartrate-resistant acid phosphatase (TRAP) staining and calcein and alizarin red labeling showed no differences in bone formation or bone resorption between the two groups (Fig. S2c–h). Likewise, the physiological bone phenotype analysis of female mice revealed no significant differences in bone development (Fig. S3a–h). These results indicate that *Irg1* deficiency does not cause any detectable defects in bone development.

**Fig. 2:**
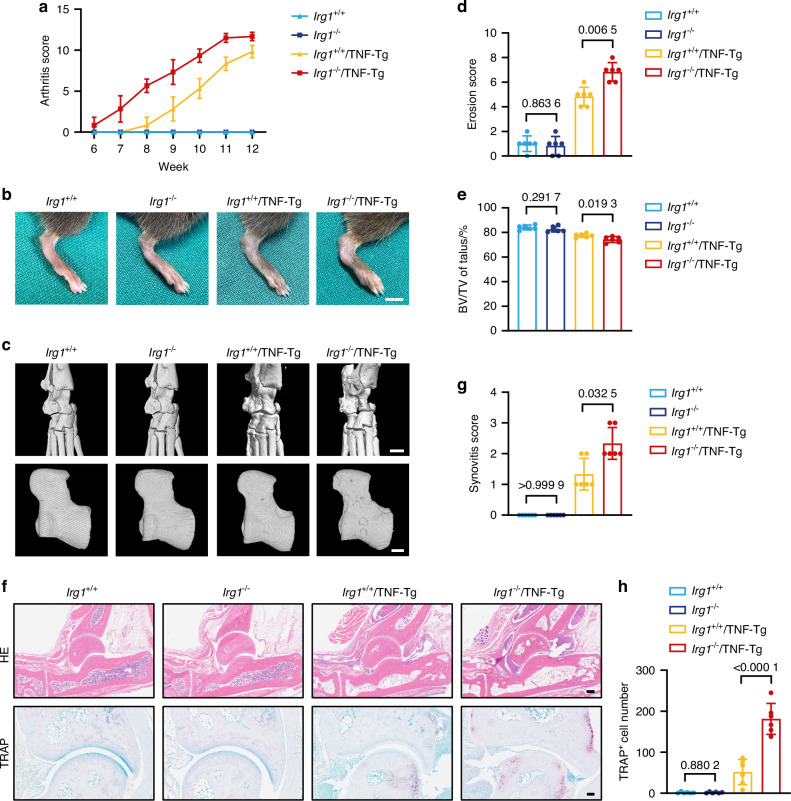
Genetic ablation of ITA-producing enzyme Irg1 induces a more severe bone destruction phenotype in TNF-Tg-induced RA mouse model. **a** Arthritis scores of *Irg1*^*+/+*^, *Irg1*^*−/−*^, *Irg1*^*+/+*^/TNF-Tg, and *Irg1*^*−/−*^/TNF-Tg mice from 6 to 12 weeks of age (*n* = 6). **b** Representative photographs of rear paws of *Irg1*^*+/+*^, *Irg1*^*−/−*^, *Irg1*^*+/+*^/TNF-Tg, and *Irg1*^*−/−*^/TNF-Tg mice (*n* = 6). Scale bar: 1 cm. **c** Representative μCT reconstruction images of the rear paws and talus of four groups (*n* = 6). Scale bar: 1 mm (top), 50 μm (bottom). **d**, **e** Erosion scores of the rear paws (**d**) and measurement of BV/TV of talus (**e**) of four groups (*n* = 6). **f** Representative images of HE staining and TRAP staining of ankle joints of *Irg1*^*+/+*^, *Irg1*^*−/−*^, *Irg1*^*+/+*^/TNF-Tg, and *Irg1*^*−/−*^/TNF-Tg mice (*n* = 6). Scale bar: 200 μm (top), 100 μm (bottom). **g** Synovitis scores based on HE staining of ankle joints (*n* = 6). **h** Quantification of osteoclast numbers around the ankle joint based on TRAP staining (*n* = 6). Significant differences were determined by Mann-Whitney *U*-test (**d**, **g**) and Student’s t-test (**e**, **h**). Data represent means ± SD for each group

Next, we employed two complementary mouse models: the collagen-induced arthritis (CIA) model, characterized by severe synovial inflammation (Fig. S4a), and the TNF-Tg model, which spontaneously develops RA due to excessive TNF-α production (Fig. S4b). In both models, ITA production was robustly upregulated (Fig. S4c, d), further supporting the notion that IRG1/ITA play key roles in the pathogenesis of RA. To investigate the role of IRG1/ITA in the pathogenesis of RA, we generated a crossbreed of TNF-Tg mice with *Irg1*-deficient mice and monitored four groups of mice—*Irg1*^*+/+*^, *Irg1*^*−/−*^, *Irg1*^*+/+*^/TNF-Tg, and *Irg1*^*−/−*^/TNF-Tg mice—for the incidence of arthritis, arthritis scores, and paw thickness starting at 6 weeks of age. Mice were sacrificed at 12 weeks, and the serum levels of ITA were measured (Fig. S5a). While there was no significant difference in RA development between *Irg1*^*+/+*^ and *Irg1*^*−/−*^ mice, *Irg1*^*−/−*^/TNF-Tg mice exhibited an earlier onset of arthritis, with visible ankle swelling by 6 weeks when compared to their *Irg1*^*+/+*^/TNF-Tg counterparts (Fig. S5b). By 12 weeks, *Irg1*^*−/−*^/TNF-Tg mice exhibited significantly higher arthritis scores, more severe hind paw swelling, and greater paw thickness (Figs. 2a, b and S5c).

Additionally, RNA sequencing of hind paw tissues identified 130 genes upregulated in *Irg1*^*−/−*^/TNF-Tg mice compared to *Irg1*^*+/+*^/TNF-Tg mice. KEGG pathway analysis indicated that these upregulated genes were predominantly associated with inflammation and osteoclast activation (Fig. S5d). Consistent with this, μCT analysis confirmed that *Irg1*^*−/−*^/TNF-Tg mice experienced more severe bone destruction than *Irg1*^*+/+*^/TNF-Tg mice (Fig. 2c–e). Histological analysis using hematoxylin and eosin (HE) and TRAP staining of the ankle joints illustrated more pronounced synovitis and an increased number of osteoclasts in *Irg1*^*−/−*^/TNF-Tg mice compared to *Irg1*^*+/+*^/TNF-Tg controls (Fig. 2f–h).

Collectively, these findings suggest that *Irg1* deficiency does not affect normal bone development but exacerbates RA-induced osteoclast formation and bone destruction.

### Itaconate, produced by inflammatory macrophages, inhibits osteoclast formation

**Fig. 3:**
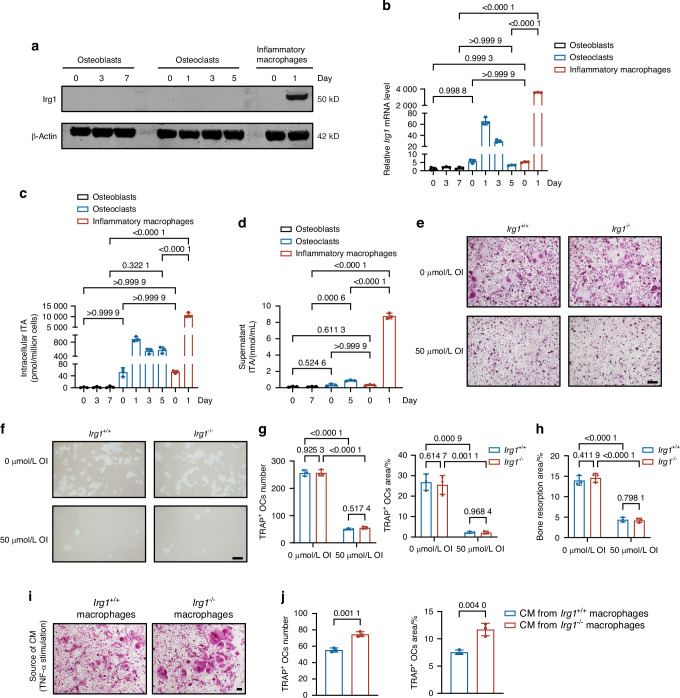
ITA derived from inflammatory macrophages inhibits osteoclast differentiation. **a** Irg1 protein levels during the differentiation of osteoblasts, osteoclasts, and inflammatory macrophages (*n* = 3). **b** *Irg1* mRNA levels during the differentiation of osteoblasts, osteoclasts, and inflammatory macrophages (*n* = 3). **c** Intracellular ITA levels during the differentiation of osteoblasts, osteoclasts, and inflammatory macrophages (*n* = 3). **d** ITA levels in cell culture supernatants during the differentiation of osteoblasts, osteoclasts, and inflammatory macrophages (*n* = 3). **e**–**h** Representative images of TRAP staining (**e**) and quantification of TRAP^+^ osteoclasts (**g**) of *Irg1*^*+/+*^ and *Irg1*^*−/−*^BMMs after 5 days of osteoclast induction with or without the addition of 50 μmol/L OI (*n* = 3). Scale bar: 10 μm. Representative images of pit formation assay (**f**) and quantification of bone resorption area (**h**) of *Irg1*^*+/+*^ and *Irg1*^*−/−*^ BMMs after 7 days of osteoclast induction with or without the addition of 50 μmol/L OI (*n* = 3). **i**, **j** Representative TRAP staining images (**i**) and quantification of TRAP^+^ osteoclasts (**j**) cultured with CM from TNF-α-stimulated *Irg1*^*+/+*^ and *Irg1*^*−/−*^ inflammatory macrophages (*n* = 3). Scale bar: 10 μm. Significant differences were determined by one-way ANOVA (**b**, **c**, **d**, **g**, **h**) and Student’s *t*-test (**j**). Data represent means ± SD for each group

To identify the primary cell type responsible for ITA production in bone tissues, we compared the intracellular levels of endogenous ITA across various cell types, including monocytes, macrophages, osteoclasts, mesenchymal stem cells (MSCs), and osteoblasts. Our data demonstrated that macrophages produced substantially higher amounts of ITA in response to LPS stimulation (Fig. S6a). In contrast, Irg1 protein expression was undetectable in MSCs or differentiated osteoblasts (Fig. 3a). While the mRNA expression of *Irg1* could be induced in osteoclastic precursor cells and mature osteoclasts, the increase—around 60-fold in response to RANKL—was relatively modest when compared to macrophages, which exhibited a robust 4000-fold upregulation of *Irg1* expression following LPS treatment (Fig. 3b). This was further supported by the observation that intracellular and extracellular ITA levels were much higher in LPS-stimulated inflammatory macrophages than in osteoclasts, which produced only minimal amounts of ITA in response to RANKL, as well as osteoblasts, in which no detectable ITA was observed (Fig. 3c, d).

In BMMs from *Irg1*^*+/+*^ and *Irg1*^*−/−*^ mice, we observed no significant differences in cell proliferation or osteoclast differentiation between the two genotypes (Fig. S6b–f). These findings indicate that the trace amounts of ITA produced by osteoclasts may not have a substantial effect on osteoclast function.

Next, we set out to investigate whether ITA produced by macrophages would affect osteoclast formation. We found that exogenous 4-octyl-itaconate (OI), a cell-permeable ITA derivative, inhibited the number and area of TRAP^+^ osteoclasts in both *Irg1*^*+/+*^ and *Irg1*^*−/−*^ cells after five days of differentiation (Fig. 3e, g). OI also reduced bone resorption activity to a similar extent in both genotypes (Fig. 3f, h). These results raise the possibility that ITA originating from macrophages may act in a paracrine manner, rather than directly influencing osteoclasts in a cell-autonomous way.

To reinforce this notion, we used conditional medium from TNF-α-treated *Irg1*^*+/+*^ and *Irg1*^*−/−*^ macrophages to stimulate osteoclast differentiation. As expected, high concentrations of ITA were present in the supernatants from *Irg1*^*+/+*^ cells, but were absent in *Irg1*^*−/−*^ cells (Fig. S7a). Interestingly, osteoclast formation was remarkably reduced in cultures treated with conditional medium from *Irg1*^*+/+*^ macrophages, while no such reduction was observed in those treated with medium from *Irg1*^*−/−*^ macrophages (Figs. 3i, j and S7b). Similar effects were obtained when using conditional medium collected from LPS-stimulated macrophages (Fig. S7a, c, d), further supporting that ITA produced by macrophages under inflammatory conditions suppresses osteoclast formation.

### Tet2 loss alleviates osteoclastic bone destruction in RA

**Fig. 4:**
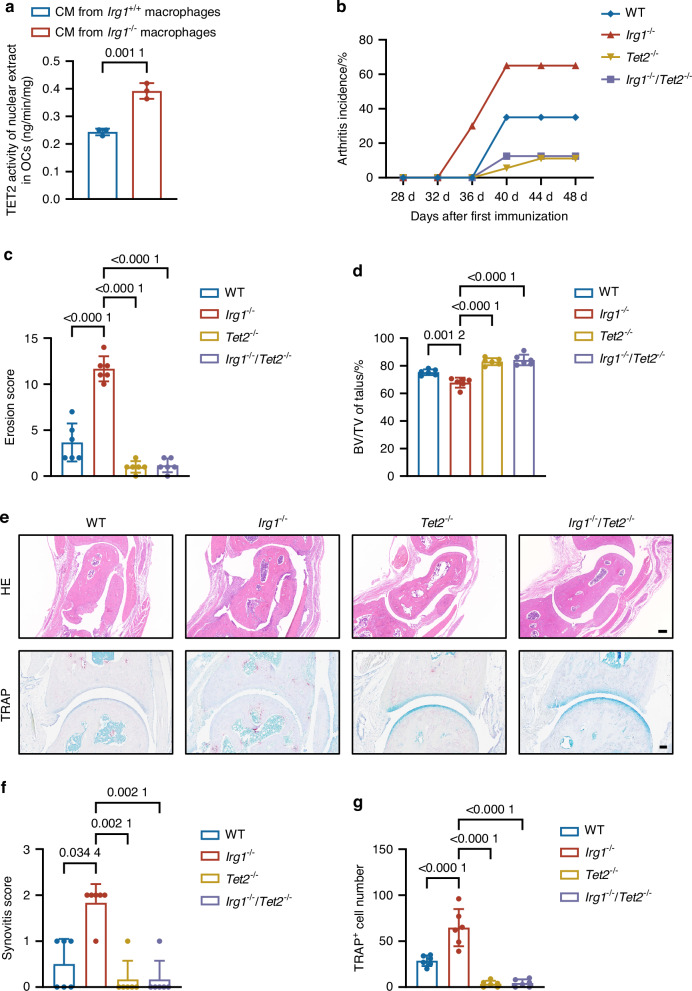
Deletion of *Tet2* reverses *Irg1* knockout-induced osteoclasts activation in CIA model. **a** Assessment of TETs activity in nuclear extracts from BMMs induced for osteoclast differentiation cultured with CM from TNF-α-stimulated *Irg1*^*+/+*^ and *Irg1*^*−/−*^ inflammatory macrophages (*n* = 3). **b** Arthritis incidence of WT (*n* = 20), *Irg1*^*−/*−^ (*n* = 20), *Tet2*^*−/−*^ (*n* = 18) and *Irg1*^*−/*−^ *Tet2*^*−/−*^ CIA mice (*n* = 16). **c**, **d** Erosion scores of the rear paws (**c**) and measurement of BV/TV (**d**) of the talus of four groups (*n* = 6). **e** Representative HE staining images and TRAP staining images of ankle joints of four groups (*n* = 6). Scale bar: 200 μm (top), 100 μm (bottom). **f** Synovitis scores based on HE staining of ankle joints (*n* = 6). **g** Quantification of osteoclast numbers around the ankle joint based on TRAP staining (*n* = 6). Significant differences were determined by Student’s *t*-test (**a**), one-way ANOVA (**c**, **d**, **g**), and Kruskal–Wallis test (**f**). Data represent means ± SD for each group

We recently identified ITA as a selective inhibitor of TET2, one of the key epigenetic enzymes responsible for catalyzing the conversion of 5-methylcytosine (5mC) to 5-hydroxymethylcytosine (5hmC) in DNA, thereby promoting DNA demethylation and changing gene expression.^21,28^ In vitro differentiation assay revealed that osteoclasts cultured in conditional medium from TNF-α-stimulated *Irg1*^*+/+*^ macrophages exhibited reduced Tet activity (Fig. 4a), as determined by a commercial TET activity assay kit.

To decipher whether Irg1/ITA-Tet2 axis plays a key role in RA development in vivo, we generated *Tet2* and *Irg1* double-knockout mice (*Irg1*^*−/*−^/*Tet2*^*−/−*^) and induced RA utilizing the standard CIA model (Fig. S8A). Consistent with previous findings,^25^ *Irg1*^*−/*−^ mice exhibited an earlier onset and higher incidence of arthritis compared with wildtype mice (Fig. 4b), alongside significantly increased bone destruction. Strikingly, *Tet2* deficiency had a protective effect against bone destruction. The exacerbated osteoclast activation and bone destruction caused by *Irg1* deficiency were significantly reversed in *Irg1*^*−/*−^/*Tet2*^*−/−*^ mice (Figs. 4c, d and S8b). In accord, histological analysis, including HE and TRAP staining of the ankle joints, revealed pronounced synovial inflammation and increased osteoclast activation in *Irg1*^*−/*−^ mice, whereas *Irg1*^*−/*−^/*Tet2*^*−/−*^ mice exhibited milder synovial inflammation and minimal osteoclast activation (Fig. 4e–g). These results clearly show Tet2 loss can rescue the excessive osteoclast activation and bone destruction phenotype caused by *Irg1* deficiency in the context of RA, thereby providing genetic evidence to support the crucial role of the IRG1/ITA-TET2 axis in osteoclast regulation and disease progression.

### ITA inhibits Tet2 to suppress osteoclast-associated genes

**Fig. 5:**
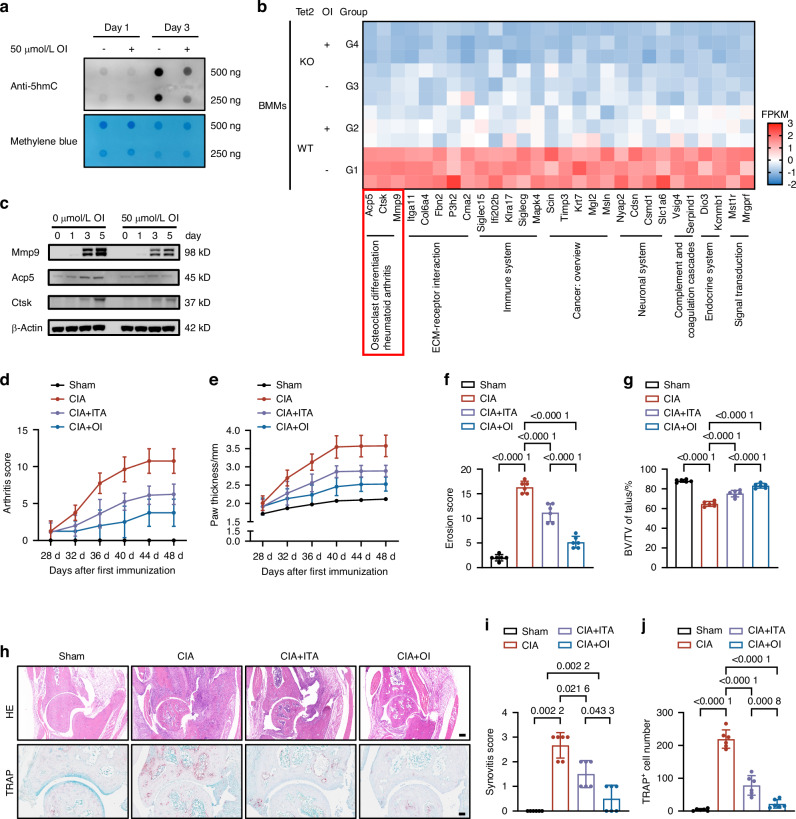
ITA alleviates arthritis and bone destruction in CIA mice by inhibiting Tet2 activity in osteoclasts. **a** DNA dot blot assay of 5hmC levels in osteoclasts treated with 50 μmol/L OI during differentiation on days 1 and 3 (*n* = 3). **b** Heatmap of relative expression of selected target genes based on FPKM (fragments per kilobase of transcript per million mapped reads) values across different groups with functional annotations. **c** Expression levels of Acp5, Ctsk, and Mmp9 proteins during osteoclast differentiation on days 0, 1, 3, and 5 with or without OI treatment (*n* = 3). **d**, **e** Arthritis scores (**d**) and rear paw thickness (**e**) of CIA mice treated with cyclodextrin, ITA, or OI (*n* = 8). **f**, **g** Erosion scores of the rear paws (**f**) and measurement of BV/TV of talus (**g**) of four groups(n = 6). **h** Representative images of HE staining and TRAP staining of 4 groups (*n* = 6). Scale bar: 200 μm (top), 100 μm (bottom). **i** Synovitis scores based on HE staining of ankle joints (*n* = 6). **j** Quantification of osteoclast numbers around the ankle joint based on TRAP staining (*n* = 6). Significant differences were determined by one-way ANOVA (**f**, **g**, **j**) and Mann–Whitney *U*-test (**i**). Data represent means ± SD for each group. **a**, **c** *n* = 3 biological independent experiments

It has been reported that ITA inhibits TET activity by competing with α-KG.^21^ DNA dot-blot assays confirmed that OI treatment reduced genomic DNA 5hmC levels in osteoclasts (Fig. 5a), indicating suppression of Tet2-mediated DNA demethylation activity. Transcriptome analysis of differentiated osteoclasts identified 58 genes that were downregulated by both OI treatment and Tet2 knockout (Fig. S9a). After excluding 21 genes that were downregulated by OI independently of Tet2, 37 genes were pinpointed as potential targets of the itaconate-Tet pathway (Fig. S9b). KEGG signaling pathway analysis of these 37 genes revealed that they are associated with RA, ECM-receptor interaction, and osteoclast differentiation (Fig. S9c).

Among these genes, *Acp5, Ctsk, and Mmp9* were widely known to play critical roles in regulating RA pathogenesis and osteoclast function (Fig. 5b).^29,30^ Hydroxymethylated DNA immunoprecipitation with qPCR (hMeDIP-qPCR) analysis demonstrated that OI robustly suppressed DNA demethylation at the promoter regions of *Acp5, Ctsk*, and *Mmp9* on the third day of osteoclast differentiation (Fig. S9d). The downregulation of Acp5, Ctsk, and Mmp9 mRNA and protein levels was confirmed by qPCR and western blot (Figs. 5c and S9e). These findings collectively indicate that OI inhibits osteoclast differentiation by suppressing TET2 activity and downregulating key osteoclast-associated genes involved in bone destruction and RA progression.

To investigate whether the inhibitory effects of OI on osteoclast differentiation were due to its competition with α-KG, a co-substrate of Tet, we treated osteoclast cultures with 50 μmol/L OI along with increasing concentrations of α-KG. Supplementation with α-KG significantly reversed the inhibitory effects of OI, as evidenced by an increase in TRAP^+^ osteoclasts and enhanced bone resorption activity, with the most pronounced effects observed at 0.5 mmol/L α-KG (Fig. S10a–e). In addition, α-KG supplementation partially restored the expression of key osteoclast differentiation genes (e.g., *Acp5, Ctsk, Mmp9)* in a dose-dependent manner (Fig. S10f). Exogenous α-KG supplementation also restored TET2 activity, as evidenced by elevated 5hmC levels (Fig. S10g, h). These findings indicate that α-KG can effectively counteract the inhibitory effects of OI on osteoclast differentiation by restoring TET2 activity and reactivating key genes involved in osteoclast function and bone resorption.

### Itaconate or its derivatives as therapeutics for RA

We next examined the potential beneficial effects of ITA and OI on cultured osteoclasts. TRAP staining showed that OI significantly inhibited osteoclast differentiation at concentrations without affecting cell viability (Fig. S11a–d), and ITA showed a similar effect on inhibiting osteoclast differentiation (Fig. S12a–d). Moreover, we found *Tet2*^*−/−*^ osteoclasts showed a significant reduction in numbers, surface areas, and bone resorption functions. The addition of OI failed to elicit any further inhibitory effects on differentiation and function in *Tet2*^*−/−*^ osteoclasts, indicating that Tet2 is indispensable for the regulatory function of OI (Fig. S13a–g). OI was more effective than ITA, probably due to its superior membrane permeability.^15^

Finally, we used DBA/1 mice with CIA to evaluate whether exogenous administration of ITA confers protective effects against RA. Compared to control mice, supplementation with ITA or OI significantly reduced hind paw swelling, arthritis incidence and arthritis scores, and decreased the thickness of the rear paws (Figs. 5d, e and S14a, b). μCT analysis revealed that supplementation with ITA or OI significantly reduced bone destruction scores in the hind paws and preserved the bone mass of the talus (Figs. 5f, g and S14c). Additionally, histological analysis confirmed that ITA or OI reduced synovial inflammation scores and decreased the number of activated osteoclasts around the ankle joints (Fig. 5h–j). We performed histological staining and analysis on the heart, liver, spleen, lungs, and kidneys of mice in the Sham, CIA, CIA + ITA, and CIA + OI groups to verify that ITA and OI showed no obvious tissue toxicity (Fig. S15).

To investigate the role of ITA in the macrophage M1-M2 shift, we observed that LPS-treated *Irg1*^*−/−*^ BMMs exhibited a significantly increased proportion of CD86^+^ cells and elevated M1/M2 ratio compared to *Irg1*^*+/+*^ cells, accompanied by a marked reduction in CD206^+^ cell proportion (Fig. S16a). RT-qPCR analysis further revealed upregulated expression of inflammation-related genes and downregulated tissue repair-associated genes in *Irg1*^*−/−*^ macrophages (Fig. S16b). OI treatment significantly reduced the proportion of CD86^+^ cells and decreased the M1/M2 ratio (Fig. S16c). RT-qPCR demonstrated that OI effectively suppressed the expression of *iNos* and *Il1b* while enhancing *Arg1* and *Cd206* expression, suggesting that OI induces partial conversion of M1 macrophages toward the M2 phenotype (Fig. S16d).

To further explore the role of OI in promoting M1-M2 shift in RA pathology, we isolated synovial tissues from the hind paws of Sham, CIA, and OI-treated CIA mice. Flow cytometry analysis revealed that OI treatment reduced CD86^+^ cell proportion and increased CD206^+^ cell proportion in synovial tissues (Fig. S16e). Additionally, we isolated peripheral blood mononuclear cells (PBMCs) from newly diagnosed RA patients in active phases and differentiated them into RA-monocyte-derived macrophages (RA-MDMs). OI-treated macrophages exhibited a more reparative phenotype (Fig. S16f, g).

Furthermore, we found that LPS-treated *Tet2*^*−/−*^ BMMs displayed a significantly reduced proportion of CD86^+^ cells and a lower M1/M2 ratio compared to *Tet2*^*+/+*^ cells, alongside a marked increase in CD206^+^ cell proportion (Fig. S17a). Quantitative analysis further demonstrated that *Tet2*^*−/−*^ macrophages exhibited downregulated expression of inflammation-associated genes (*iNos*, *Il1b*) and upregulated tissue repair-related genes (*Arg1*, *Cd206*) (Fig. S17b).

Taken together, these results demonstrate that exogenous administration of ITA or OI provides substantial protection against arthritis by reducing inflammation, osteoclast activation, and bone destruction. Additionally, ITA induces M1-M2 shift in macrophages, accompanied by RA alleviation.

## Discussion

In contrast to the moderate induction of Irg1 expression by RANKL in osteoclasts, TNF-α robustly induces Irg1 expression in macrophages, thereby exerting a strong inhibitory effect on osteoclast differentiation. This suggests the presence of an intrinsic balancing mechanism within the body, where ITA production and secretion from macrophages help counteract excessive osteoclast activation, thereby mitigating bone destruction in the inflammatory environment driven by TNF-α. To the best of our knowledge, our study is the first to reveal an intercellular mechanism by which macrophage-derived ITA in the bone tissue microenvironment can efficiently enter osteoclasts and inhibit their differentiation. These findings provide new insights into the role of macrophages in RA, underscoring the critical involvement of macrophage-derived ITA in the disease’s pathogenesis and progression. Moreover, our research highlights ITA as a key mediator facilitating the crosstalk between macrophages and osteoclasts. The role of ITA in RA has recently garnered significant attention. One study reported that RANKL stimulation induces ITA production in osteoclasts, and Irg1 knockout in these cells reduces ITA levels, thereby enhancing osteoclast formation.^25^ However, another independent study found no significant differences in osteoclast differentiation between *Irg1*^+/+^ and *Irg1*^−/−^ BMMs.^26^ Consistent with the latter study, *Irg1* deficiency did not affect osteoclast differentiation. Furthermore, no significant differences in bone mass or osteoclast formation were observed between *Irg1*^+/+^ and *Irg1*^−/−^ mice under physiological conditions. This lack of effect may be attributed to the relatively weak induction of Irg1 expression by RANKL in osteoclasts, resulting in insufficient endogenous ITA levels to inhibit osteoclast differentiation.

Epigenetic alterations, such as DNA methylation and histone acetylation, play crucial roles in the onset and pathogenesis of RA by influencing the post-transcriptional expression of key genes involved in synovial inflammation and bone destruction.^31,32^ Recent studies have highlighted the effect of epigenetic factors, including DNA methyltransferase (DNMT), Lysine-Specific Demethylase 1 (LSD1), and Histone Deacetylase (HDAC), in regulating osteoclastogenesis in RA.^33–35^ Nevertheless, therapeutic strategies targeting these epigenetic modifications have yet to be validated in RA patients. An emerging concept in autoimmune disease treatment involves the use of metabolites to therapeutically reprogram immune cells through epigenetic modifications. For example, butyrate has been shown to mitigate inflammatory bowel disease by modulating the epigenetic landscape of neutrophils and attenuate primary biliary cholangitis by epigenetic reprogramming of myeloid-derived suppressor cells.^36,37^ Studies have shown that Nicotinamide adenine dinucleotide (NAD^+^) can treat allergic diseases by epigenetically modifying mast cells, and its supplementation can modulate the Th1 and Th17 responses to alleviate experimental autoimmune encephalomyelitis (EAE) through the SIRT1 pathway.^38,39^ In this study, using Tet2 and Irg1 double-knockout mice, we provide genetic evidence that Irg1/ITA can regulate osteoclast differentiation through epigenetic mechanisms involving Tet2. Specifically, ITA inhibits Tet2 activity to downregulate the expression of osteoclast marker genes, such as *ACP5*, *CTSK*, and *MMP9*, thereby suppressing osteoclast differentiation. This suggests that epigenetic reprogramming of pathogenic osteoclasts in RA via ITA could represent a novel therapeutic strategy. In the occurrence and progression of RA, the complex interplay of various cells and inflammatory factors leads to chronic inflammation. Regulating inflammatory factors such as ILs plays a crucial role in restoring immune homeostasis in RA.^12,13^ ITA and TET2 have been shown to regulate the production of various cytokines, including the secretion of TNF-α, IL-1β, and IL-18, and we previously reported that ITA suppresses inflammatory responses in macrophages by inhibiting TET2 activity.^28,40,41^ These results suggest that ITA and TET2 can not only regulate the excessive activation of osteoclasts, but also impact the inflammatory microenvironment in RA through their effects on key inflammatory mediators.

To date, numerous studies have reported the therapeutic benefit of ITA or OI in animal models of various inflammatory diseases, including RA. One study reported that OI treatment mitigated inflammation and inflammation-mediated bone loss in experimental arthritis by inhibiting aerobic glycolysis of osteoclasts.^25^ Another study claimed ITA treatment ameliorated CIA by inhibiting mitochondrial oxidative phosphorylation and glycolysis of FLS.^42^ They explained the mechanisms of OI or ITA treatment for RA primarily from the perspective of energy metabolism. Our study, however, demonstrates that OI specifically regulates Tet2 to inhibit osteoclast differentiation in RA from the angle of epigenetic modification. In addition, we treated CIA mice with both ITA and OI, and compared their therapeutic effects. Exogenous supplementation with large amounts of ITA or the ITA derivative OI shows significant therapeutic effects on synovial inflammation and bone destruction in RA, with OI exhibiting superior efficacy compared to ITA. The polar carboxyl group of ITA may hinder its transmembrane transport, whereas OI, a membrane-permeable derivative, enhances lipophilicity through esterification, facilitating more efficient cellular uptake and activity.^22^ In addition, we found that ITA can promote the M1-M2 shift in macrophages, which not only serves as an effective strategy to prevent RA progression but also constitutes an important characteristic of the remission phase of RA.

TNF-α neutralizing antibodies such as infliximab, adalimumab, and etanercept are widely used for the treatment of RA. By blocking TNF-α, these antibodies reduce inflammation, slow the progression of joint damage, and alleviate symptoms such as pain and swelling. However, while effective, they may not be suitable for all patients due to potential side effects, including an increased risk of infections, and loss of efficacy over time due to the production of autoantibodies.^43^ Alternatively, itaconate represents a novel therapeutic approach by regulating immune metabolism and epigenetic modification pathways.^19^ Itaconate, being an endogenous metabolite, is presumed to have higher safety and fewer side effects. Additionally, current RA drugs such as TNF-α inhibitors and IL-6 inhibitors primarily function by blocking specific inflammatory pathways.^44,45^ In contrast, itaconate can regulate multiple immune and inflammatory responses, potentially benefiting patients who do not respond well to traditional therapies. Furthermore, itaconate and its derivatives can be used in combination with other RA medications, possibly enhancing therapeutic effects.

## Materials and methods

### Human samples

Informed consent from all patients providing peripheral blood samples or synovial fluid was obtained. The analysis of human materials was approved by the Ethics Committee of Shanghai Ninth People’s Hospital, Shanghai Jiao Tong University School of Medicine (SH9H-2024-T246-1). The patients with RA fulfilled the 2010 ACR/EULAR criteria for RA.^46^ Peripheral blood samples were obtained from the Department of Laboratory Medicine at Shanghai Ninth People’s Hospital. Synovial fluid samples were collected from the Orthopedic Specimen Bank at Shanghai Ninth People’s Hospital. These included RA patients diagnosed by the Department of Rheumatology and referred to the Department of Orthopedics for knee replacement surgery, as well as OA patients admitted to the Department of Orthopedics for knee replacement surgery. Demographics and clinical features of patients involved in the collection are listed in Tables S1–S3.

### Mice

*Tet2*^−/−^ (JAX stock no. 023359) and *Irg1*^−/−^ (JAX stock no. 029340) mice were purchased from the Jackson Laboratory. TNF-Tg (Cyagen stock no. C001250) mice were purchased from Cyagen. The animals were backcrossed onto C57BL/6J (Cyagen stock no. C001089) backgrounds, respectively, for more than seven generations. DBA/1J mice were purchased from GemPharmatech (stock no. N000219). All mice were used for analysis regardless of sex. All mice were housed in pathogen-free conditions with constant ambient temperature (22 °C ± 2 °C) and humidity (55% ± 10%), with an alternating 12-h light/dark cycle. All mice were euthanized by carbon dioxide overdose followed by cervical dislocation. All animal studies were approved by the Institutional Animal Care and Use Committee at Ninth People’s Hospital, School of Medicine, Shanghai Jiao Tong University (SH9H-2024-A1288-1).

### CIA model and ITA/OI intervention

CIA model was performed according to a previously published protocol.^47^ Briefly, 8-week-old DBA/1J mice were immunized intradermally at the base of the tail with 100 μg of Chicken type II collagen (Chondrex,20012) emulsified in complete Freund’s adjuvant (Chondrex, 7009) in equal volumes. Twenty-one days later, a booster immunization was performed using 100 μg of Chicken type II collagen in incomplete Freund’s adjuvant (Chondrex, 7002). For 8-week-old C57BL/6J mice, both the initial and booster immunizations were administered subcutaneously, using 200 μg of Chicken type II collagen emulsified in complete Freund’s adjuvant (Chondrex, 7023).^48^ Clinical score was assessed after the booster immunization using the following system detailed previously: 0, normal; 1, mild, but definite redness and swelling of the ankle or wrist, or apparent redness and swelling limited to individual digits, regardless of the number of affected digits; 2, moderate redness and swelling of ankle or wrist; 3, Severe redness and swelling of the entire paw including digits; 4, maximally inflamed limb with involvement of multiple joints. The scores of all four limbs were summed, yielding total scores of 0 to 16 per mouse. Regarding the treatment of CIA animals, Itaconate (Sigma, I29204) and OI (MCE, HY-112675) was first dissolved in DMSO (100 mg/mL) (MCE, HY-Y0320) and further diluted with 40%((2-Hydroxypropyl)-β-cyclodextrin) in PBS to a final concentration of 10 mg/mL. DBA/1 mice were administered OI at 100 mg/kg or vehicle every other day by i.p. injection.

### μCT analysis

μCT analysis of fixed tibia or rear paw was conducted using a Skyscan 1176 (Bruker) at 50 kV, 100 μA, and a resolution of 9 μm. The acquired images were reconstructed with NRecon softwarev1.7 (Bruker). The region from 50 to 250 slices below the growth plate was analyzed for BV/TV, Tb.Th, Tb.N, and Tb.Sp using the program CTAn v1.16 (Bruker). Six sites in the ankle joint were evaluated: the talus, navicular bone, medial cuneiform bone, and the bases of the first, second, and third metatarsals. Each site was rated on a scale from 0 to 3 (0 = normal; 1 = pitting; 2 = full-thickness holes in small to medium areas; and 3 = full-thickness holes in medium to large areas), with a maximum total score of 18. The final erosion score for the arthritic hind paw was obtained by averaging the scores assigned by two observers.^49^ We used ankles for μCT analysis because, in human patients with RA, official radiographic quantification of bone erosion (Sharp score) is typically performed on the wrists or ankles.

### Histological staining and immunofluorescence staining

For hard tissue sections, the mice were intraperitoneally injected with 8 mg/kg calcein solution at 10 weeks of age and 20 mg/kg alizarin red at 11 weeks of age. At 12 weeks, the mice were euthanized, and their femurs were harvested. The femurs were not decalcified and were embedded in methyl methacrylate (MMA) resin.^50^ Sections were cut to a thickness of 5 μm using a Leica RM2255 microtome (Leica) and used for Von Kossa and TRAP staining. Blank sections were utilized for the analysis of double fluorescence labeling. For decalcified paraffin-embedded sections, the tissue was first fixed in 4% paraformaldehyde. After routine fixation, the samples were decalcified in 10% EDTA (pH 7.4) for 2 weeks. Following decalcification, the samples were processed for paraffin embedding and were then cut into sections with a thickness of 4 μm using a Leica RM2255 microtome. HE and TRAP staining were performed according to the manufacturer’s protocols for histological evaluation of the ankl joints in mice.

### In vitro differentiation of macrophages and osteoclasts

BMMs were extracted by flushing the femurs and tibias of 4 to 6-week-old WT, *Irg1*^*−/−*^, or Tet2^*−/−*^ mice on a C57BL/6J background and cultured in alpha-modified Eagle’s medium (α-MEM, Hyclone, SH30265.01) containing 10% FBS (Hyclone, SH30406.05) and 1% penicillin-streptomycin (NCM Biotech, C100C5). Cell viability was maintained with 50 ng/mL recombinant mouse M-CSF (SinoBiological, 51112-MNAH). For macrophage activation, BMMs were cultured in complete medium supplemented with 100 ng/mL LPS (Sigma, L2880) or 100 ng/mL recombinant TNF-α (SinoBiological, 50349-MNAE). For osteoclast induction, BMMs were cultured in complete medium containing 50 ng/mL recombinant mouse M-CSF and 100 ng/mL recombinant mouse RANKL (SinoBiological, 50343-M01H).

For all the cultures, media was changed every two days until indicated timepoints. For the TRAP staining experiment, BMMs were seeded at a density of 8 000 cells/well in a 96-well plate and induced to differentiate into osteoclasts for five days. The cells were then stained with Tartrate-Resistant Acid Phosphatase Dye (JoyTech, 1-0002) and photographed. For the bone resorption assay, BMMs were seeded at a density of 8 000 cells/well on Osteo Assay Surface plates (Corning, 3988) or bovine bone slices (JoyTech, 2-0002) and induced to differentiate into osteoclasts for seven days. Images were captured using a bright-field microscope or scanned with a scanning electron microscope.

### Human monocytes culture

Human monocytes were isolated PBMCs of RA patients by density gradient centrifugation using Ficoll-Paque^TM^ PLUS (Cytiva, 17-1440-02) and overnight adhesion in 12-well plates in RPMI 1640 medium (Hyclone, SH30809.01) containing 10% FBS and 1% penicillin-streptomycin. Cultured monocytes were treated with Phorbol 12-myristate 13-acetate (PMA, MCE, HY-18739) 5 ng/mL for 24 h to induce their differentiation into RA-MDMs. Afterwards, cultured RA-MDMs were treated with or without 100 μmol/L OI for 12 h for RT–qPCR analysis or 24 h for flow cytometry analysis.

### Flow cytometry

Mouse synovial tissues were isolated from knee joints, digested with 1 mg/mL type I collagenase (Sigma, C0130) in HBSS (Gibco, C14175500BT), and incubated at 37 °C and 5% CO_2_ under gentle shaking for 1 h. Disaggregated tissue elements were passed through a 70 µm cell strainer. Single-cell suspensions from mouse tissues and in vitro culture were stained with Fixable Viability Stain 780 (FVS780, BD Biosciences, 565388) and indicated fluorochrome-conjugated antibodies against cell-surface markers including Brilliant Violet 421™ anti-mouse F4/80 Antibody (BioLegend, 123131), Brilliant Violet 510™ anti-mouse CD45 Antibody (BioLegend, 157219), FITC anti-mouse/human CD11b Antibody (BioLegend, 101205), PE Rat Anti-Mouse CD86 (BD Biosciences, 553692), Brilliant Violet 421™ anti-mouse/human CD11b Antibody (BioLegend, 101235) and FITC anti-human CD86 Antibody (BioLegend, 374203). For staining intracellular markers, including Alexa Fluor® 647 Rat Anti-Mouse CD206 (BD Biosciences, 565250) and APC anti-human CD206 (MMR) Antibody (BioLegend, 321109), the Cytofix/Cytoperm™ Fixation and Permeabilization Solution (BD Biosciences, 554722) was used after surface marker staining. Data were acquired using Cytek^®^ Aurora (Cytek Biosciences) and analyzed using FlowJo v10.9.0 (BD Biosciences).

### RNA-sequencing

Total RNA from osteoclasts differentiated for three days and mouse hind paw tissues was extracted and purified with the RNeasy Mini Kit (QIAGEN, 74104). Sequencing was performed at BGI and Majorbio. Differential gene expression analysis between groups was conducted using DESeq2,^51,52^ with criteria of Fold Change ≥ 2 and Adjusted *P* value ≤ 0.001, depending on the project. For between-sample differential gene analysis, PoissonDis was used with conditions of Fold Change ≥ 2 and FDR ≤ 0.001. A heatmap of differentially expressed gene clusters was generated using the heatmap function. Based on GO and KEGG annotations, differentially expressed genes were functionally classified. KEGG enrichment analysis was performed using the phyper function in R, and GO enrichment analysis was done with the TermFinder package. Candidate genes with a Q value ≤ 0.05 were considered significantly enriched.

### Single-cell RNA-seq data processing

Raw data consisting of 314 011 cells were downloaded from Synapse (10.7303/syn52297840).^27^ Quality control and preprocessing were performed using established bioinformatics tools to ensure data integrity. Following this, data normalization, dimensionality reduction, and clustering analyzes were conducted referencing the strategy outlined in the original publication. Differential expression analysis and functional enrichment analysis were employed to identify key genes and pathways, shedding light on biological processes and cellular heterogeneity. The results were visualized using various visualization tools to provide comprehensive insights into the data. The results published here are in whole or in part based on data obtained from the ARK Portal (arkportal.synapse.org).

### Metabolomics

The metabonomic analysis of synovial fluid was performed by Metabo-Profile Biotechnology Co. Ltd, China, using the commercial Q300 Kit (Metabo-Profile) following the standard processing protocol.^53^ Internal standards were added to the derivatized samples, which were then randomly analyzed and quantitated using an ultra-performance liquid chromatography coupled to tandem mass spectrometry (UPLC-MS/MS) system. The raw data generated were processed using the QuanMET v2.0.

### LC–MS/MS and GC-TOFMS

The cell and cell supernatant extracts were analyzed using an ultra-high performance liquid chromatograph (Acquity UPLC I-Class, Waters) coupled to a triple quadrupole mass spectrometer (Xevo TQ-XS, Waters). C13-labeled ITA was added as an internal standard. The data were analyzed using MassLynx v4.2. Serum and synovial fluid were analyzed by a gas chromatograph (7890, Agilent) coupled to a time-of-flight mass spectrometer (Pegasus 4D, LECO). The data were processed with ChemStation vC.01.07.

### RNA purification and RT–qPCR

Total RNA from cells was extracted and purified using the RNeasy Mini Kit (QIAGEN). RNA was reverse transcribed using the PrimeScript™ RT Reagent Kit (TaKaRa, RR037). The diluted complementary DNA (cDNA) was used in RT-qPCR reactions containing TB Green® Premix Ex Taq™ (TaKaRa, RR420) and gene-specific primers. The reactions were performed on a QuantStudio 6 Flex real-time PCR system (Applied Biosystems). β-Actin was used as a housekeeping control. The primers are listed in Table S4.

### Western blot

Cells were collected and lysed in RIPA buffer (Beyotime, P0013C) supplemented with protease inhibitors. Protein samples were subjected to separated on 4%–20% ExpressPlus™ PAGE Gel (GenScript, M42015C), transferred to PVDF membranes (Millipore, ISEQ00010), and blocked with 5% non-fat milk. Overnight incubation was performed at 4 °C with primary antibodies: IRG1 antibody (1:1 000, Abcam, ab222411), MMP9 antibody (1:1 000, Abcam, ab228402), Cathepsin K antibody (1:1 000, Abcam, ab37259), ACP5 antibody (1:1 000, Affinity, DF6989), and β-actin antibody (1:1 000, Affinity, T0022). Following primary antibody incubation, the membranes were probed with secondary antibodies for detection: Mouse IgG, HRP-linked antibody (1:5 000, CST, 7076) and Rabbit IgG, HRP-linked antibody (1:5 000, CST, 7074). Imaging was performed using e-BLOT Touch Imager (eBLOT).

### ELISA

Peripheral blood from humans was centrifuged at 2 000 × *g* for 30 min, and the serum was collected and stored at −80 °C for subsequent experiments. Human CTX-1(Elabscience, E-EL-H0835), human TNF-α (Elabscience, E-EL-H0109), and human IL-6 (Elabscience, E-EL-H6156) were measured according to the manufacturer’s protocols.

### DNA dot-blot

The DNA dot blot assay was conducted as previously described with some modifications.^54,55^ Briefly, genomic DNA was extracted from osteoclasts and denatured at 95 °C for 10 min. The DNA was then spotted onto a nitrocellulose membrane (Whatman). The membrane was subjected to UV light for 30 min to cross-link the DNA and subsequently stained with 0.03% methylene blue solution. After blocking with 5% non-fat milk in TBS–Tween 20 for 1 h, the membrane was incubated overnight at 4 °C with 5hmC antibody (1:500, Active Motif, 39769). Following incubation with Rabbit IgG, HRP-linked antibody (1:5 000, CST, 7074) for 1 h at room temperature, the membrane was scanned using a Typhoon scanner (GE Healthcare).

### TET2 in vitro activity assay

The Epigenase 5mC-Hydroxylase TET Activity/Inhibition Assay Kit (Epigenase, P-3086-96) was used following the manufacturer’s protocol to assess TET2 enzyme activity. Briefly, 4 μg of nuclear extracts in a 4 μL volume were added to a 50 μL total reaction mixture. After incubating at 37 °C for 90 min, the absorbance was measured at 450 nm.

### hMeDIP-qPCR

Genomic DNA was extracted from the samples using a standard phenol-chloroform extraction method as described.^56^ Genomic DNA was sonicated (320 W, 5 min), denatured at 95 °C for 10 min, and immunoprecipitated with 2 μg/mL 5hmC antibody (Active Motif, 39769) or 2 μg/mL Rabbit IgG, monoclonal [EPR25A]—Isotype Control (Abcam, ab172730) overnight at 4 °C with gentle rotation. Subsequently, 50 µL of Pierce Protein G agarose (Thermo Fisher, 20399) was added to the antigen-antibody complex and incubated with gentle rotation for 2 h at room temperature. After washing with PBS, immune complexes were eluted with 100 µL of 0.2 mol/L glycine HCl buffer (pH 3.0) and neutralized with 10 µL of 1 mol/L Tris (pH 8.0). DNA was then extracted using QIAquick PCR Purification Kit (QIAGEN, 28004) and analyzed by qPCR. The primers used are listed in Table S3.

### Statistical and reproducibility

**Fig. 6:**
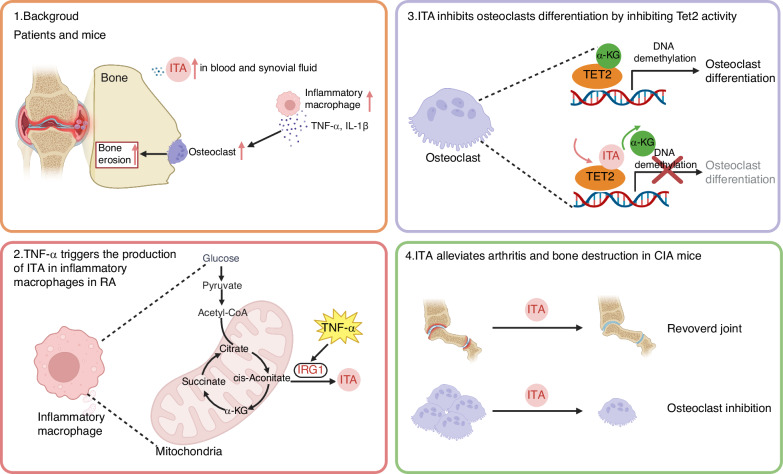
Schematic summary of TNF-α-driven macrophage-derived ITA in suppressing RA-associated osteoclast hyperactivation through Tet2 inhibition

All statistical analyzes were performed using GraphPad Prism software v8.0 (GraphPad Software) and are presented as mean ± standard deviation (SD). Comparisons between two groups were made using unpaired two-tailed *t*-test or paired two-tailed *t*-test, while comparisons among three or more groups were made using Kruskal–Wallis test, one-way analysis of variance (ANOVA), or two-way ANOVA. Statistical results are indicated by *P* values in the figures, and *P* < 0.05 was considered statistically significant. Unless otherwise stated, all experiments were repeated in triplicate or more (Fig. 6).

## Supplementary information

Inflammatory Macrophage-derived Itaconate Inhibits DNA Demethylase TET2 to Prevent Excessive Osteoclast Activation in Rheumatoid Arthritis

## Data Availability

The datasets used and/or analyzed during the current study are available from the corresponding author upon reasonable request. The raw sequencing data of all samples have been uploaded to the SRA public database of the National Center for Biotechnology Information (NCBI). The accession numbers are PRJNA1245747 and PRJNA1246247.
