# Supplementary material for: Inflammatory macrophage-derived itaconate inhibits DNA demethylase TET2 to prevent excessive osteoclast activation in rheumatoid arthritis
Source: Bone Res. 2025 Jun 11;13:60. doi: 10.1038/s41413-025-00437-w (PMC12159140; doi:10.1038/s41413-025-00437-w)
Supplement: Supplementary file 1 — Inflammatory Macrophage-derived Itaconate Inhibits DNA Demethylase TET2 to Prevent Excessive Osteoclast Activation in Rheumatoid Arthritis [file 41413_2025_437_MOESM1_ESM.docx]

**Supplementary information**

**Inflammatory Macrophage-derived Itaconate Inhibits DNA Demethylase TET2 to Prevent Excessive Osteoclast Activation in Rheumatoid Arthritis**

Kewei Rong^†^, Dezheng Wang^†^, Xiting Pu^†^, Cheng Zhang, Pu Zhang, Xiankun Cao, Jinglin Zheng, Xiao Yang, Kexin Liu, Lei Shi, Yin Li, Peixiang Ma, Dan Ye, Jie Zhao^*^, Pu Wang^*^, An Qin^*^

*Correspondence:

An Qin: [dr_qinan@163.com](mailto:dr_qinan@163.com)

Pu Wang: wangpu@fudan.edu.cn

Jie Zhao: profzhaojie@126.com

†These authors contributed equally to this work.

This file includes:

Table S1 to S4

Figure. S1 to S17

**Table S****1. Demographics and clinical features of patients involved in the collection of synovial fluid samples**

| Study cohort | OA group | RA group |
| --- | --- | --- |
| Subjects, n | 15 | 15 |
| Age (years) | 65.53 ± 6.04 | 61.00 ± 5.43 |
| Female,n (%) | 80 | 93.33 |
| CRP (mg/L) | 2.66 ± 2.98 | 29.42 ± 10.86 |
| ESR (mm/h) | 11.00 ± 5.25 | 46.67 ± 22.48 |

CRP, C-reactive protein; ESR, erythrocyte sedimentation rate; csDMARDs; Continuous variables are expressed as mean ± standard deviation (SD).

**Table S2. Demographics and clinical features of patients involved in the collection of serum samples**

| Study cohort | Healthy control group | RA active group | RA remission group |
| --- | --- | --- | --- |
| Subjects, n | 15 | 21 | 18 |
| Age(years) | 50.67 ± 11.13 | 52.43 ± 13.13 | 52.33 ± 9.38 |
| Female, n (%) | 66.67 | 71.42 | 77.78 |
| CRP (mg/L) | / | 42.24 ± 27.14 | 2.00 ± 2.44 |
| ESR (mm/h) | / | 45.10 ± 19.79 | 9.50 ± 3.98 |
| DAS28-CRP | / | 4.45 ± 1.07 | 1.44 ± 0.47 |
| Treatment | none | none | csDMARDs(Methotrexate) |

CRP, C-reactive protein; ESR, erythrocyte sedimentation rate; csDMARDs, conventional synthetic Disease-Modifying Antirheumatic Drugs; DAS28-CRP, disease activity score 28; Continuous variables are expressed as mean ± standard deviation (SD).

**Table S3. Demographics and clinical features of patients involved in the collection of PBMCs**

| Study cohort | RA patients newly diagnosed and with active disease |
| --- | --- |
| Subjects, n | 13 |
| Age(years) | 52.69 ± 13.54 |
| Female, n (%) | 100 |
| CRP (mg/L) | 52.46 ± 28.73 |
| ESR (mm/h) | 61.23±21.88 |

CRP, C-reactive protein; ESR, erythrocyte sedimentation rate; Continuous variables are expressed as mean ± standard deviation (SD).

**Table S4. Primers sequences used for Real-Time PCR and hMeDIP-qPCR**

Primers for murine Real-Time PCR

| Gene name | Forward sequence | Reverse sequence |
| --- | --- | --- |
| *β-Actin* | TGTCCACCTTCCAGCAGATGT | AGCTCAGTAACAGTCCGCCTAGA |
| *Acp5* | CGGCTACTTGCGGTTTCA | CCTTGGGAGGCTGGTCTTA |
| *Ctsk* | TAGCCACGCTTCCTATCCGA | CCTCCGGAGACAGAGCAAAG |
| *Mmp9* | GGACCCGAAGCGGACATTG | CGTCGTCGAAATGGGCATCT |
| *Dcstamp* | ACCTAAGCGGAACTTAGACACA | TAGGGCTTCGTGGAAACACA |
| *Atp6v0d2* | GCAGAGCTGTACTTCAATGTGG | TAGTCCGTGGTCTGGAGATG |
| *Calcr* | GCTGTGTTTACCGACGAGCA | TCAGCCAGCAGTTGTCGTT |
| *Irg1* | AGTTTTCTGGCCTCGACCTG | AGAGGGAGGGTGGAATCTCT |
| *Tet2* | CCACGTGGCCAAAGTAAACAG | TTGAATGAATCCAGCAGCACC |
| *Il1b* | TCGCAGCAGCACATCAACAAGAG | AGGTCCACGGGAAAGACACAGG |
| *iNos* | ACTCAGCCAAGCCCTCACCTAC | TCCAATCTCTGCCTATCCGTCTCG |
| *Arg1* | GGCAACCTGTGTCCTTTCTCCTG | GGTCTACGTCTCGCAAGCCAATG |
| *Cd206* | TGATTGGTGGCAATTCACGAGAGG | AACAGGCAGGGAAGGGTCAGTC |

Primers for hMeDIP-qPCR

| *Acp5-hMeDIP-qPCR-1* | CACATGATGGGGGCTTGCTA | GCTGGGACTTCTGAGGCATT |
| --- | --- | --- |
| *Acp5-hMeDIP-qPCR-2* | AGCGGCAATAGGCAGTCAAA | TCTCTCACCTGGATGGGTGG |
| *Ctsk-hMeDIP-qPCR-1* | CCCCCAAAGTCAGTCAGATG | GGTAAGGATTGCGGAAGTCA |
| *Ctsk-hMeDIP-qPCR-2* | CTAGCCTTTCCTCCCCTCTC | CCCTAGTTGTCTCCATTCTTCC |
| *Mmp9-hMeDIP-qPCR-1* | AGGAAGGTGTTTGCCCCATC | AACAGACCCTTTGTCGCTCA |
| *Mmp9-hMeDIP-qPCR-2* | TCCACGTAAACAGCTGGGTG | GAGGAGATGTTGGCTGTCCC |

Primers for human Real-Time PCR

| Gene name | Forward sequence | Reverse sequence |
| --- | --- | --- |
| *β-Actin* | CACCATTGGCAATGAGCGGTTC | AGGTCTTTGCGGATGTCCACGT |
| *IL1B* | CCACAGACCTTCCAGGAGAATG | GTGCAGTTCAGTGATCGTACAGG |
| *NOS2* | GCTCTACACCTCCAATGTGACC | CTGCCGAGATTTGAGCCTCATG |
| *CD163* | CCAGAAGGAACTTGTAGCCACAG | CAGGCACCAAGCGTTTTGAGCT |
| *CD206* | AGCCAACACCAGCTCCTCAAGA | CAAAACGCTCGCGCATTGTCCA |

**
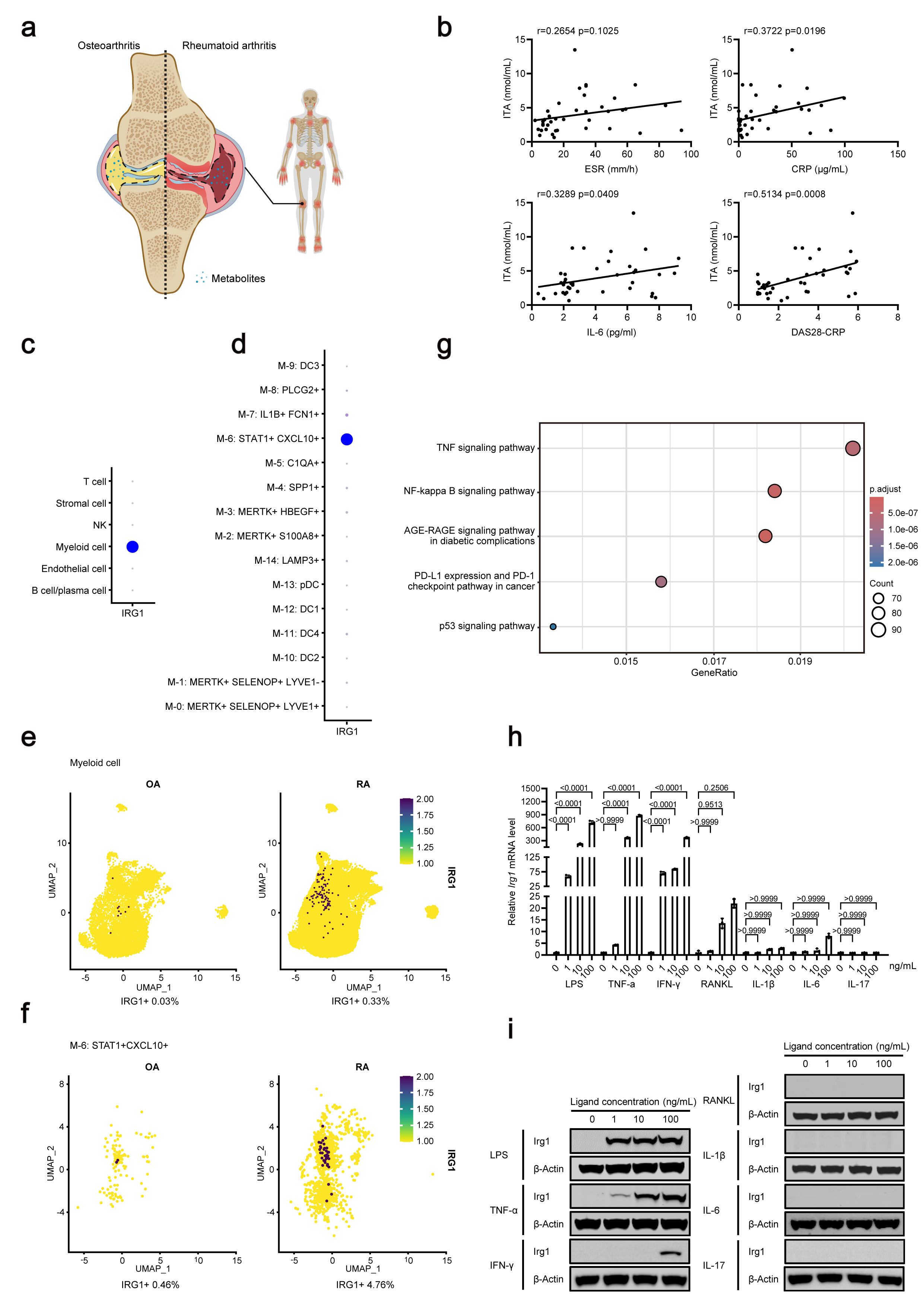
Fig. S1 TNF triggers the expression of Irg1 in inflammatory macrophages in rheumatoid arthritis. a** Illustration of ITA levels in synovial fluid of OA and RA patients. **b** Correlation analysis between blood ITA levels and blood ESR, CRP, IL-6 and DAS28-CRP levels in RA active group and RA remission group (n=39). **c** Expression of the IRG1 gene in various subtypes of cells (314 011 cells). **d** Expression of the IRG1 gene in various subtypes of myeloid cells. **e** Proportion of *IRG1^+^* cells among myeloid cells in OA and RA patients. **f** Proportion of *IRG1^+^* cells among *STAT1^+^* *CXCL10^+^* myeloid cells in OA and RA patients. **g** KEGG pathway analysis of differentially expressed genes in *IRG1^high^* and *IRG1^low^* myeloid cells. **h** Expression levels of *Irg1* mRNA in mouse BMMs stimulated with various inflammatory factors at different concentration gradients for 24 hours (n=3). **i** Expression levels of Irg1 protein in mouse BMMs stimulated with various inflammatory factors at different concentration gradients for 24 hours (n=3). Significant differences were determined by Pearson correlation (**b**) and one-way ANOVA (**h**). Data represent means ± SD for each group. **h**, **i** n=3 biological independent experiments.

**
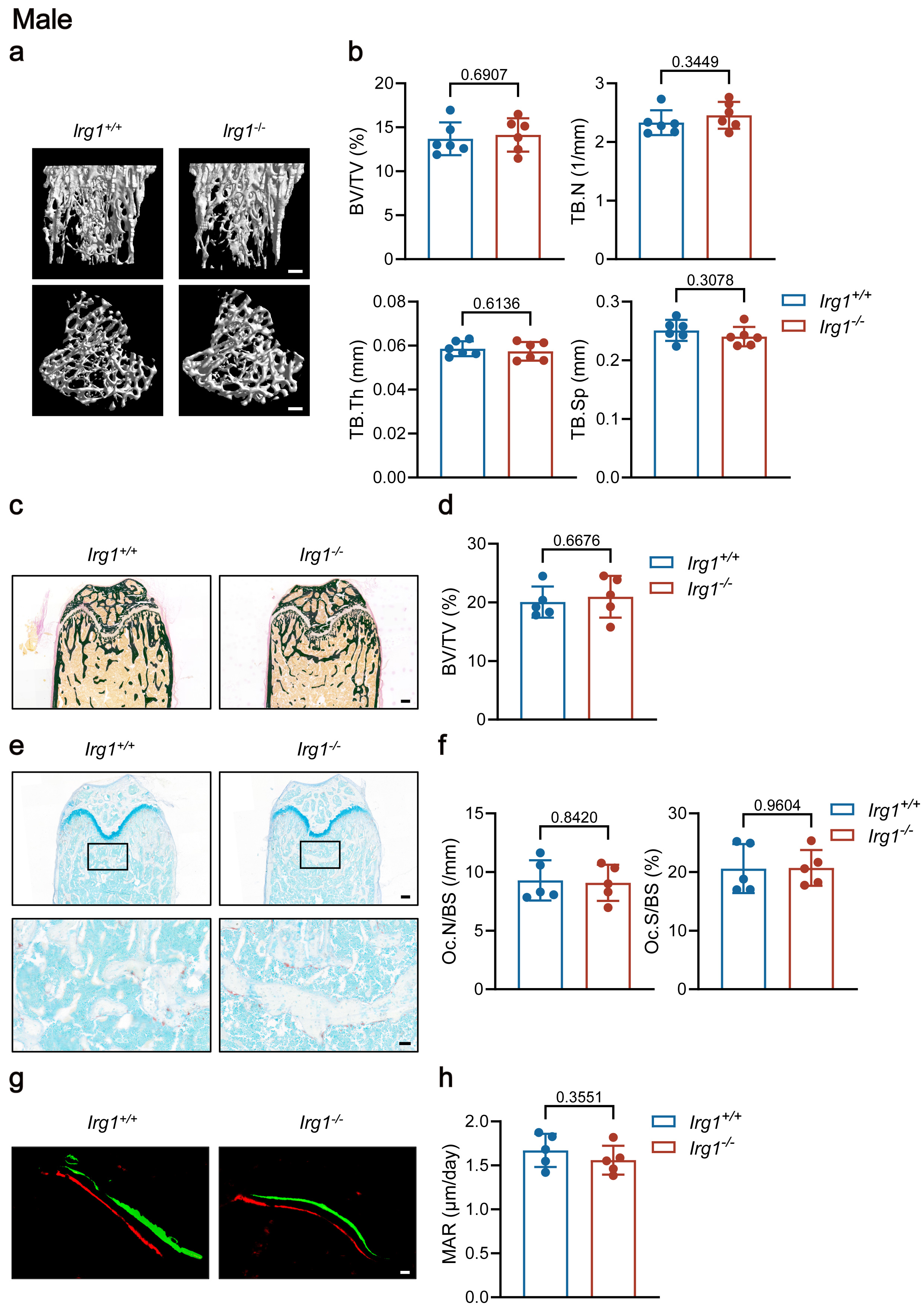
Fig. S2 *Irg1^+/+^* and *Irg1^-/-^* male mice exhibit no differences in physiological osteogenic and osteoclastic phenotypes in vivo. a** Representative tibial μCT images of the trabecular bone from 12-week-old *Irg1^+/+^* and *Irg1^-/-^* male mice (n=6). Scale bar: 200 μm. **b** Quantification of tibial Cancellous BV/TV, Tb.N, Tb.Th and Tb. Sp in 12-week-old *Irg1^+/+^* and *Irg1^-/-^* male mice (n=6). **c,d** Representative images of Von Kossa staining (**c**) and corresponding quantification of BV/TV of femurs (**d**) from 12-week-old *Irg1^+/+^* and *Irg1^-/-^* male mice (n=5). Scale bar:200 μm. **e,f** Representative images of TRAP staining (**e**) and quantification of Oc.N/BS and Oc.S/BS (**f**) of femurs from 12-week-old *Irg1^+/+^* and *Irg1^-/-^* male mice (n=5). Scale bar:200 μm (top),50 μm (bottom). **g, h** Representative images of calcein and alizarin red labeling (**g**) and quantification of MAR (**h**) of femurs from 12-week-old *Irg1^+/+^* and *Irg1^-/-^* male mice (n=5). Scale bar:10μm. Significant differences were determined by Student’s t-test (**b**, **d**, **f**, **h**). Data represent means ± SD for each group.


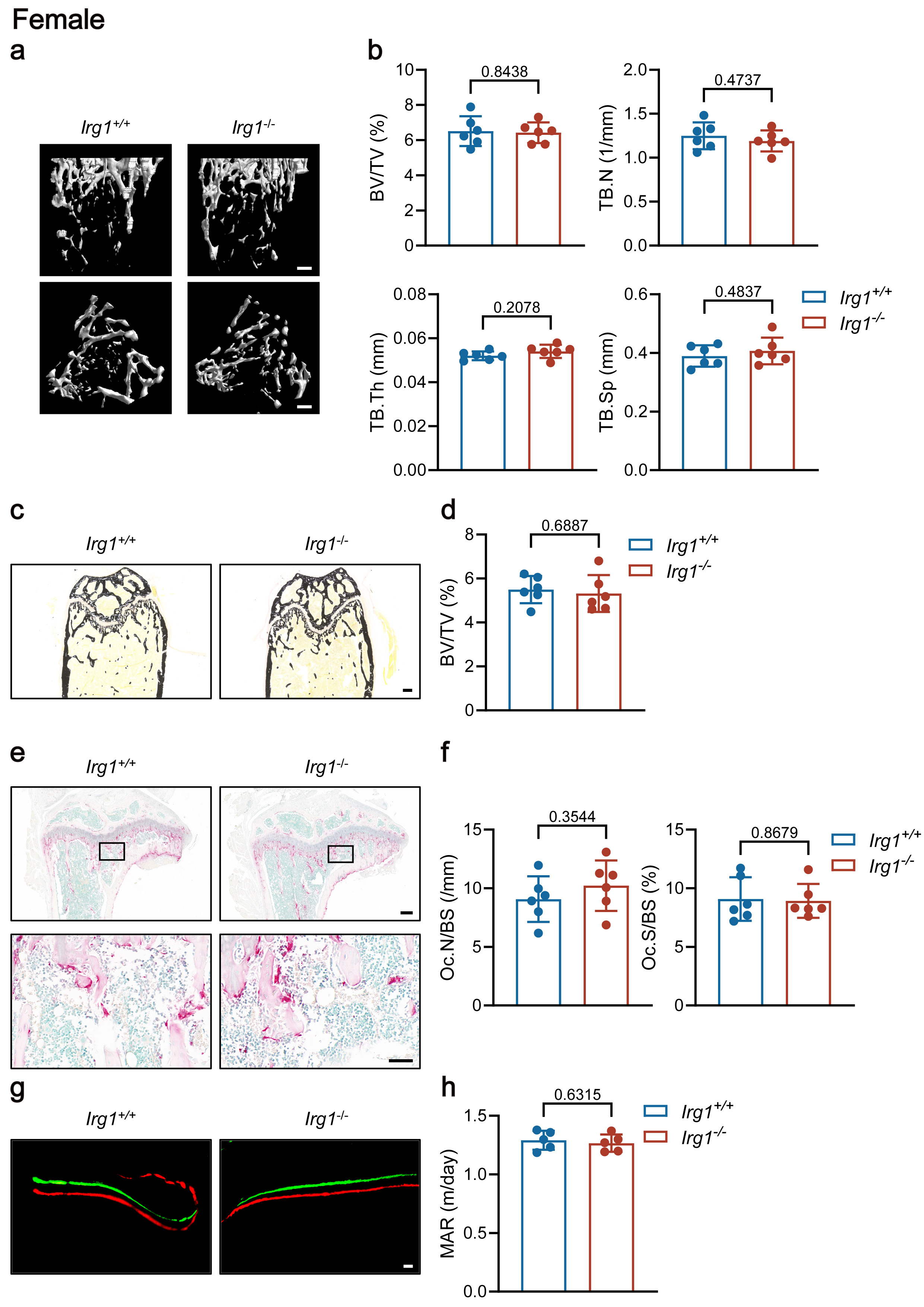
**Fig. S3 *Irg1^+/+^* and *Irg1^-/-^* female mice exhibit no differences in physiological osteogenic and osteoclastic phenotypes in vivo. a** Representative tibial μCT images of the trabecular bone from 12-week-old *Irg1^+/+^* and *Irg1^-/-^* female mice (n=6). Scale bar: 200μm. **b** Quantification of tibial Cancellous BV/TV, Tb.N, Tb.Th and Tb. Sp in 12-week-old *Irg1^+/+^* and *Irg1^-/-^* female mice (n=6). **c**, **d** Representative images of Von Kossa staining (**c**) and corresponding quantification of BV/TV (**d**) of femurs from 12-week-old *Irg1^+/+^* and *Irg1^-/-^* female mice (n=5). Scale bar:200 μm. **e**, **f** Representative images of TRAP staining (**e**) and quantification of Oc.N/BS and Oc.S/BS (**f**) of tibiae from 12-week-old *Irg1^+/+^* and *Irg1^-/-^* female mice (n=6). Scale bar:200 μm (top),50 μm (bottom). **g**, **h** Representative images of calcein and alizarin red labeling (**g**) and quantification of MAR (**h**) of femurs from 12-week-old *Irg1^+/+^* and *Irg1^-/-^* female mice (n=5). Scale bar:10 μm. Significant differences were determined by Student’s t-test (**b**, **d**, **f**, **h**). Data represent means ± SD for each group.

**
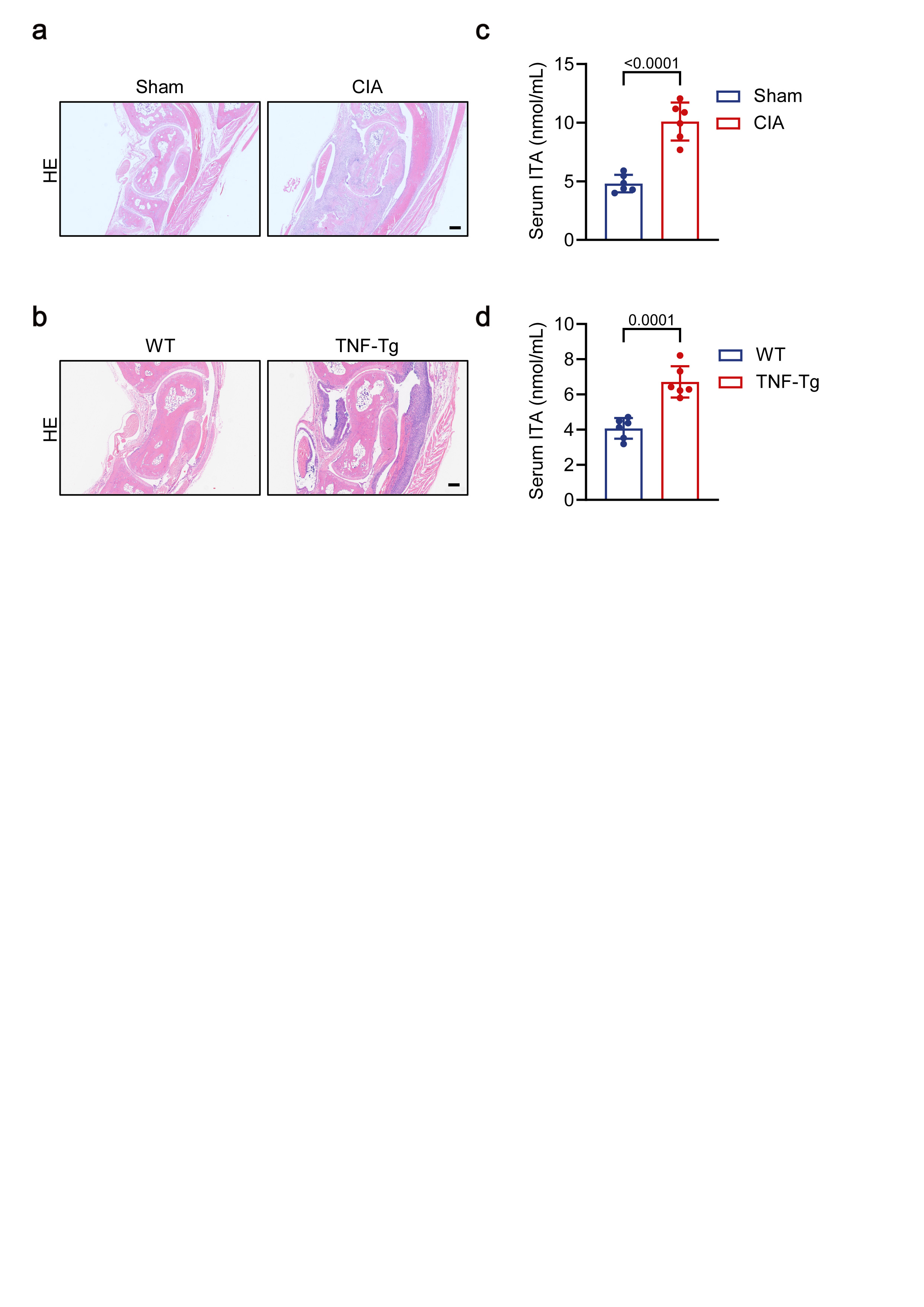
Fig. S4 Histological and quantitative analysis of ankle joints and serum ITA levels in mouse models of RA. a** Representative images of HE staining of ankle joints of Sham and CIA mice (n=6). Scale bar: 200 μm. **b** Representative images of HE staining of ankle joints of WT and TNF-Tg mice (n=3). Scale bar: 200 μm. **c** Quantitative detection of serum ITA in Sham and CIA mice(n=6). **d** Quantitative detection of serum ITA in WT and TNF-Tg mice (n=6). Significant differences were determined by Student’s t-test (**c**, **d**). Data represent means ± SD for each group.

**
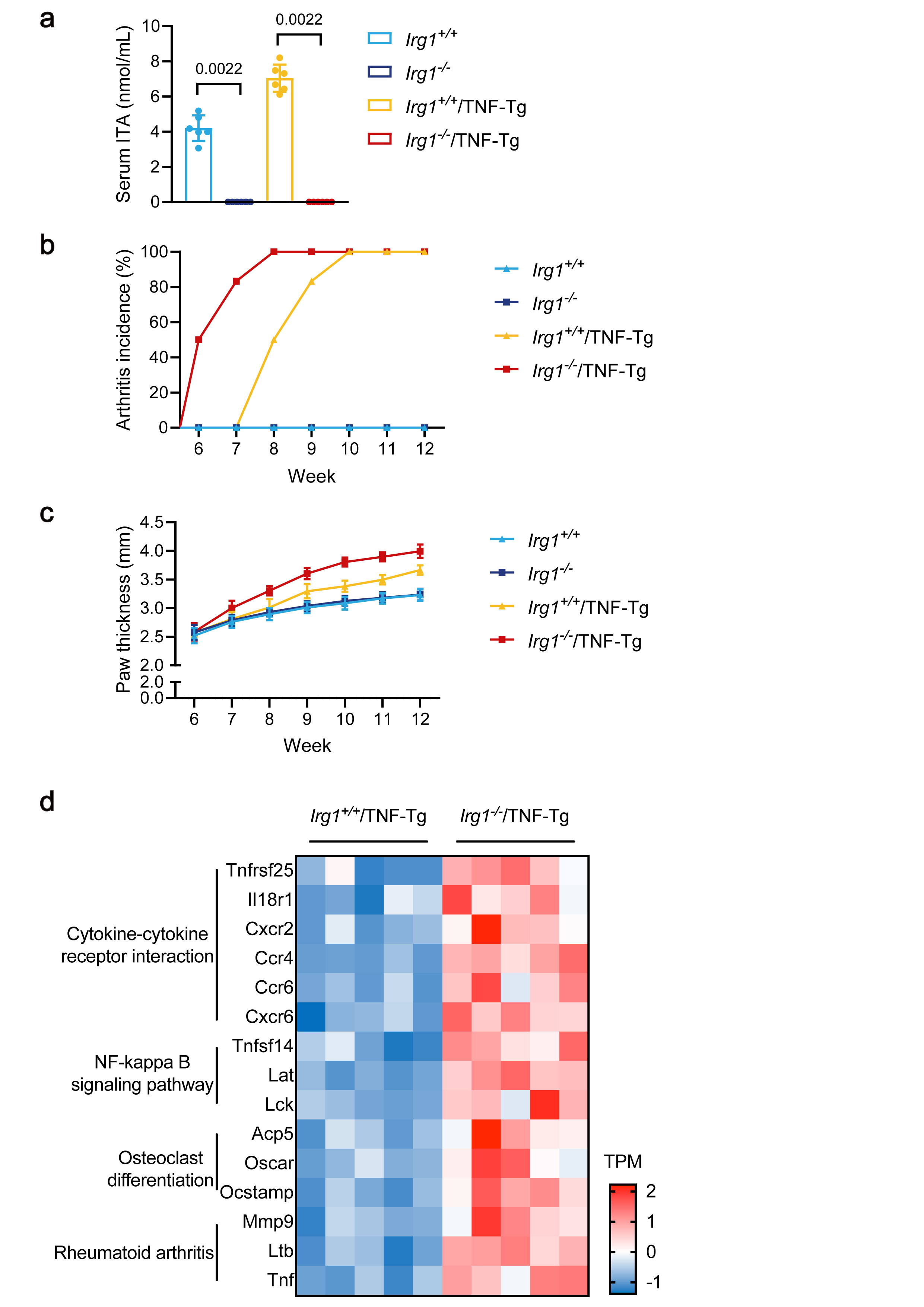
Fig. S5 Phenotypic analysis of TNF-Tg and IRG1 knockout hybrid mice.** **a** Measurement of serum ITA levels in *Irg1^+/+^*, *Irg1^-/-^*, *Irg1^+/+^*/TNF-Tg, and *Irg1^-/-^*/TNF-Tg mice (n=6). **b**, **c** Arthritis incidence (**b**) and rear paw thickness (**c**) of *Irg1^+/+^*, *Irg1^-/-^*, *Irg1^+/+^*/TNF-Tg, and *Irg1^-/-^*/TNF-Tg mice from 6 weeks to 12 weeks of age (n=6). **d** Heatmaps of representative differential genes between *Irg1^+/+^*/TNF-Tg, and *Irg1^-/-^*/TNF-Tg mice based on TPM (n=5). Significant differences were determined by Mann-Whitney U test (**a**). Data represent means ± SD for each group.


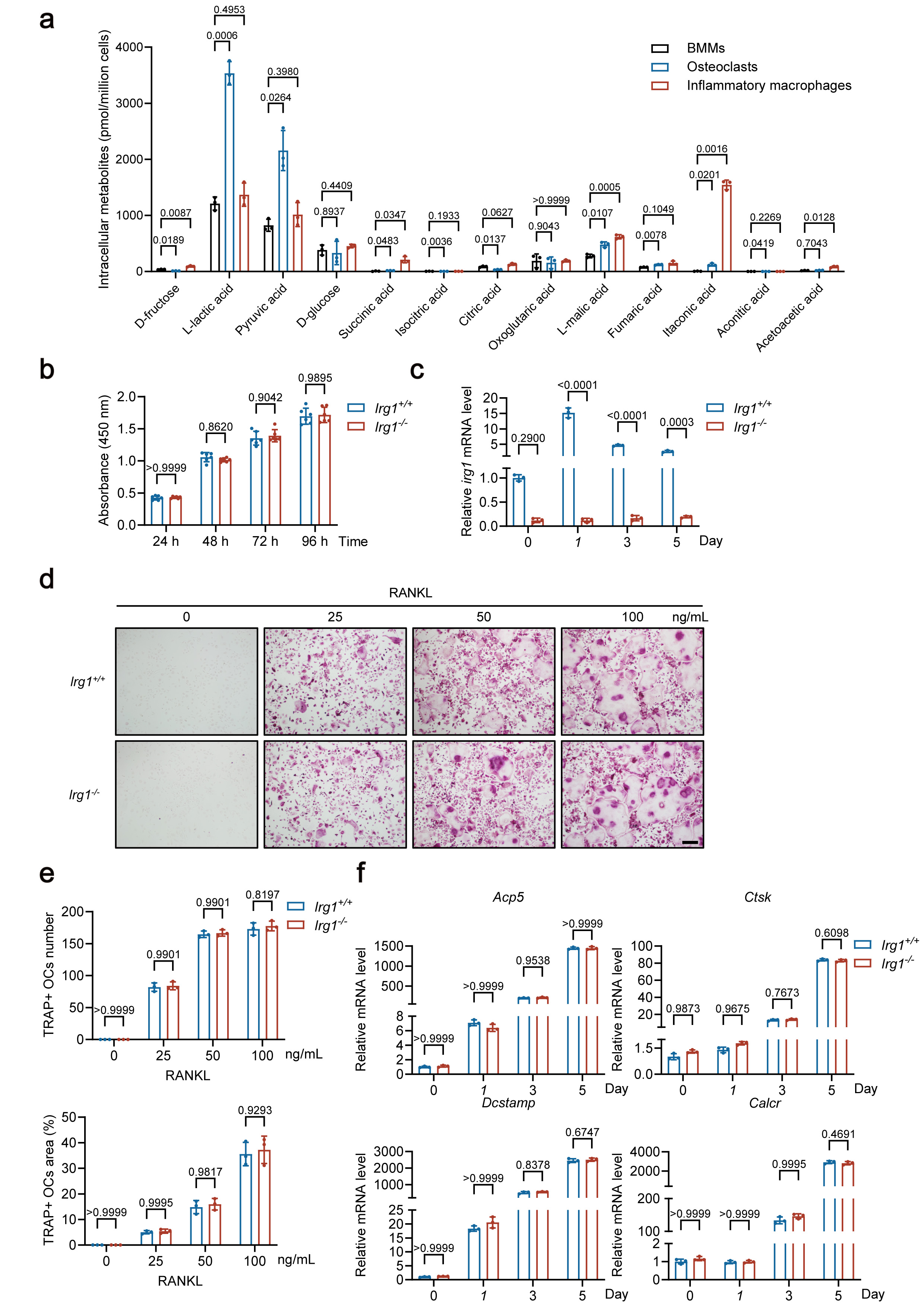
**Fig. S6 There is no significant difference in osteoclast differentiation between *Irg1^+/+^* and *Irg1^-/-^* BMMs. a** Various organic acid metabolites levels in BMMs, osteoclasts, and inflammatory macrophages (n=3). **b** CCK-8 assay of *Irg1^+/+^* and *Irg1^-/-^* BMMs cell viability at 0, 24, 48, 72, and 96 hours (n=6). **c** Expression levels of *Irg1* mRNA in *Irg1^+/+^* and *Irg1^-/-^* BMMs during osteoclast differentiation at day 0,1,3 and 5 (n=3). **d**, **e** Representative TRAP staining images (**d**) and quantification of TRAP^+^ osteoclasts (**e**) of *Irg1^+/+^* and *Irg1^-/-^* BMMs after 5 days of stimulation with gradient concentrations of RANKL (n=3). Scale bar: 10 μm. **f** Expression of osteoclast-associated genes in *Irg1^+/+^* and *Irg1^-/-^* BMMs during osteoclast differentiation at day 0,1,3and 5 (n=3). Significant differences were determined by two-way ANOVA (**a**, **b**, **c**, **e**, **f**). Data represent means ± SD for each group. **b**-**f** n=3 biological independent experiments.

**
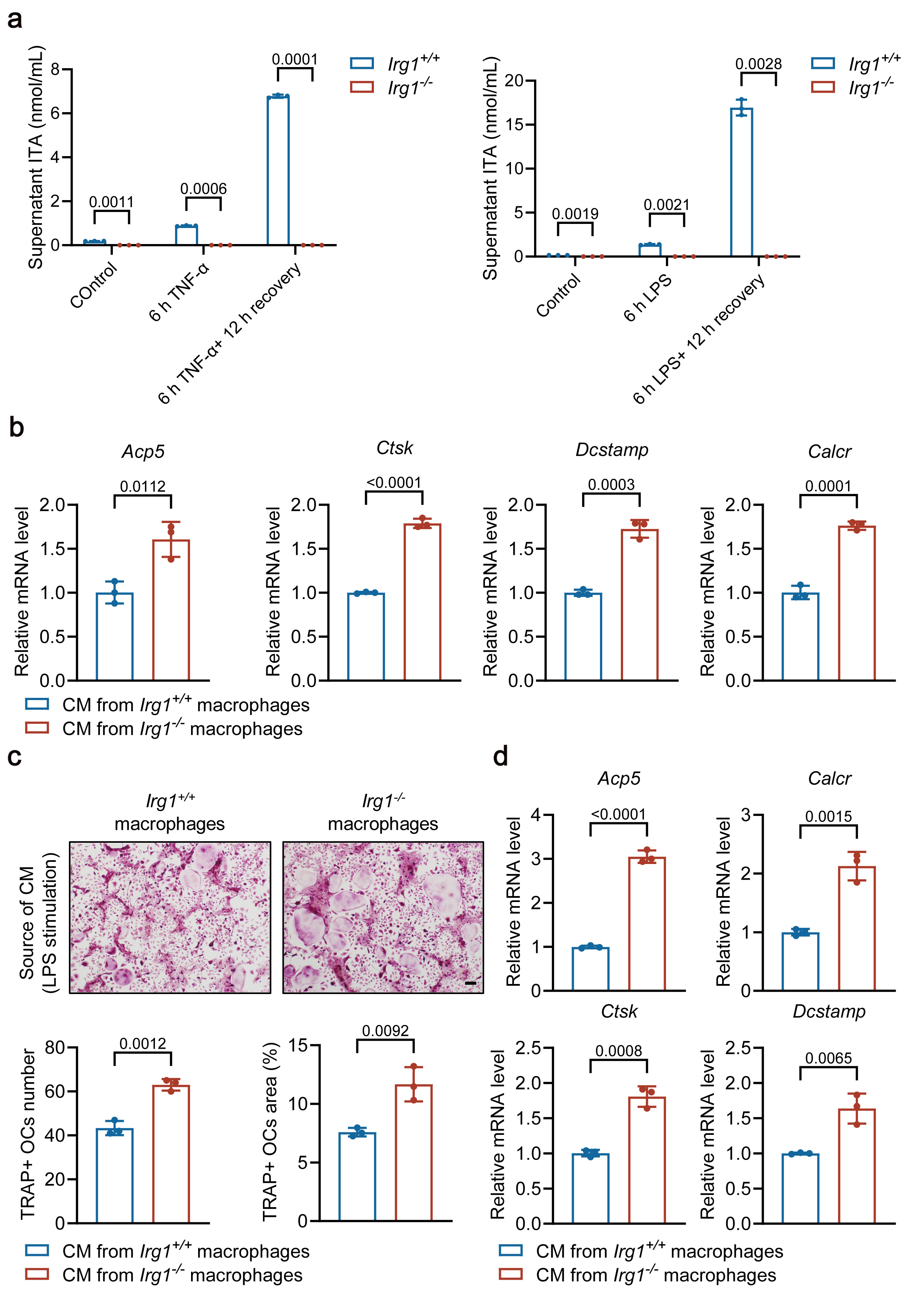
Fig. S7 ITA produced by inflammatory macrophages under TNF-α or LPS stimulation inhibits osteoclast differentiation. a** ITA levels in cell supernatants of *Irg1^+/+^* and *Irg1^-/-^* macrophages under various treatment conditions (n=3). **b** Expression of osteoclast-associated genes in osteoclasts cultured with CM from TNF-α-stimulated *Irg1^+/+^* and *Irg1^-/-^* inflammatory macrophages (n=3). **c** Representative TRAP staining images and quantification of TRAP^+^ osteoclasts cultured with CM from LPS-stimulated *Irg1^+/+^* and *Irg1^-/-^* inflammatory macrophages (n=3). Scale bar: 10 μm. **d** Expression of osteoclast-associated genes in osteoclasts cultured with CM from LPS-stimulated *Irg1^+/+^* and *Irg1^-/-^* inflammatory macrophages (n=3). Significant differences were determined by two-way ANOVA (**a**) and Student’s t-test (**b**, **c**, **d**). Data represent means ± SD for each group. **b**, **c**, **d** n=3 biological independent experiments.

**
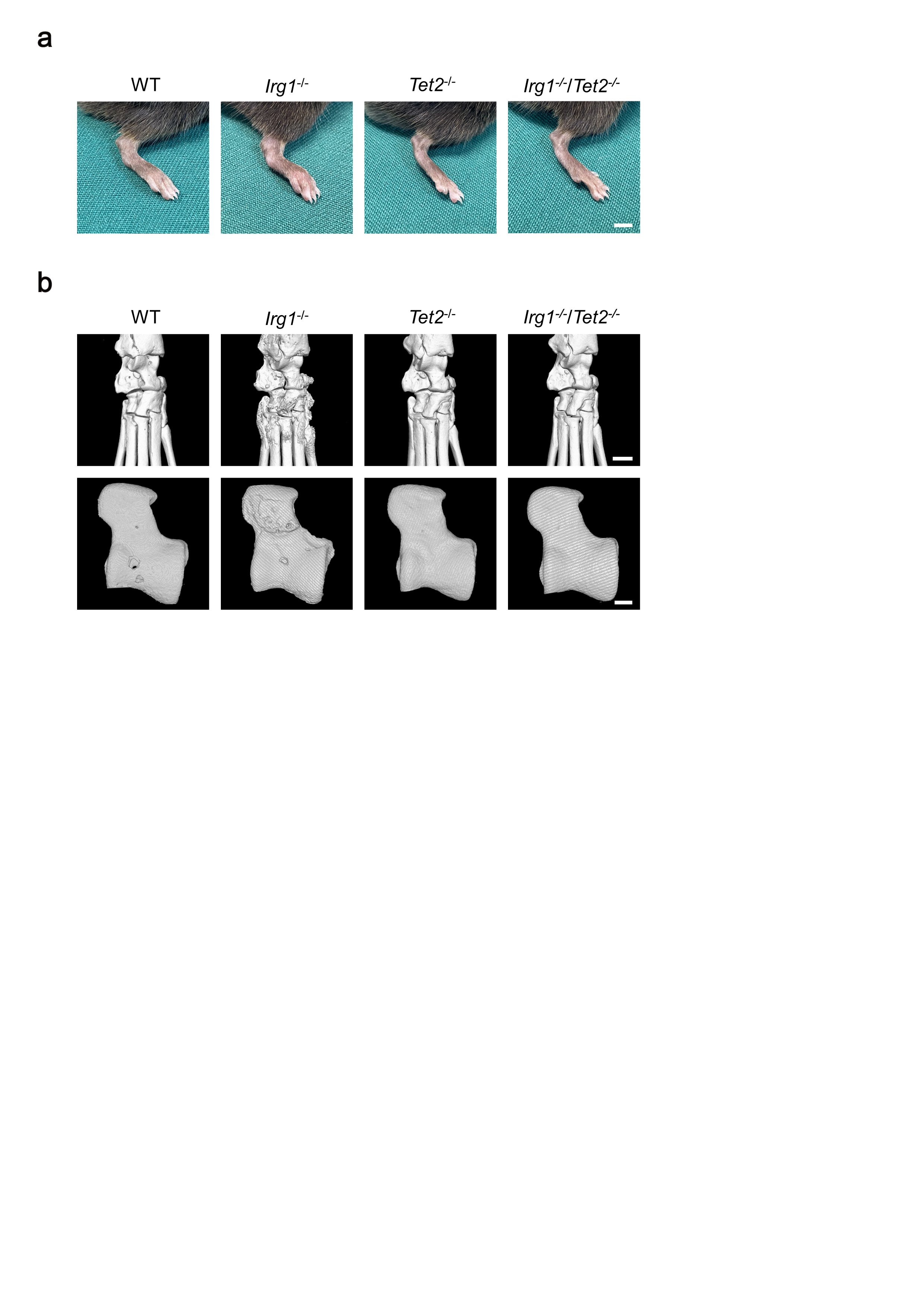
Fig. S8 Representative photographs and μCT reconstruction images of rear paws in WT, *Irg1^-/^*^-^, *Tet2^-/-^* and *Irg1^-/^*^-^/*Tet2^-/-^* mice. a** Representative photographs of the CIA mice rear paws from WT, *Irg1^-/^*^-^, *Tet2^-/-^* and *Irg1^-/^*^-^/*Tet2^-/-^* mice (n=8). Scale bar:1 cm. **b** Representative μCT reconstruction images of rear paws and talus of WT, *Irg1^-/^*^-^, *Tet2^-/-^* and *Irg1^-/^*^-^/*Tet2^-/-^* mice (n=6). Scale bars: 1mm (top), 100μm (bottom).

**
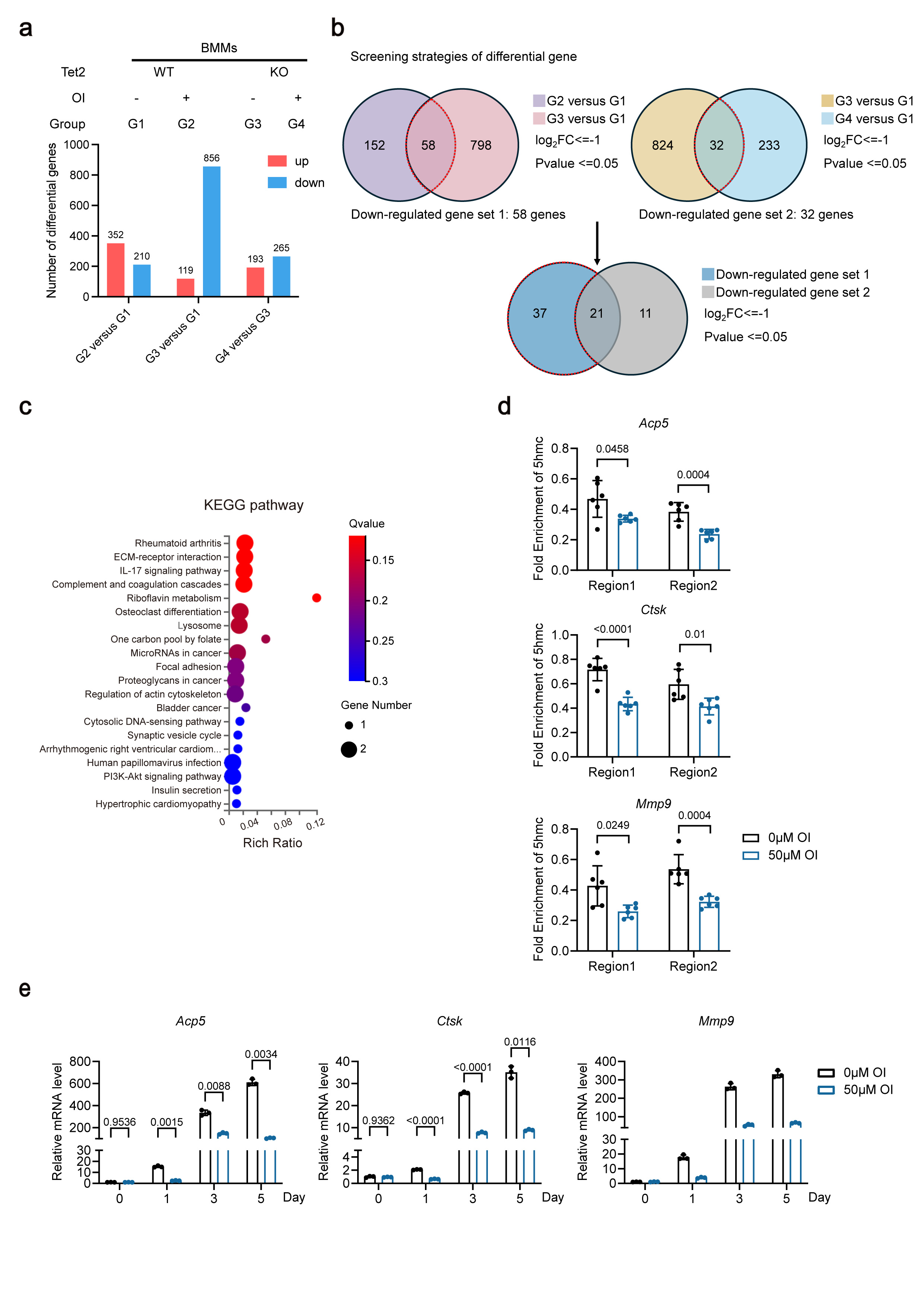
Fig. S9 OI inhibits the demethylation of the promoter regions of Acp5, Ctsk, and Mmp9. a** RNA-seq analyses of the effect of OI treatment and *Tet2* deletion on the regulation of gene expression in MCSF and RANKL-treated BMMs cells. Grouping and number of differentially expressed genes between different groups (n=3). **b** Screening strategy for identifying 37 genes strictly regulated by tet2 among OI-inhibited osteoclast differentiation genes. **c** The top twenty pathways enriched among the overlapping 37 genes identified by KEGG pathway analysis. **d** Promoter demethylation Status of *Acp5*, Ctsk, and *Mmp9* following three days of osteoclastogenesis in the presence or absence of OI (n=6). **e** Expression levels of *Acp5*, *Ctsk*, and *Mmp9* mRNA during osteoclast differentiation at days 0, 1, 3, and 5 with or without OI treatment (n=3). Significant differences were determined by Student’s t-test (**d**) and two-way ANOVA (**e**). Data represent means ± SD for each group. **d**, **e** n=3 biological independent experiments.


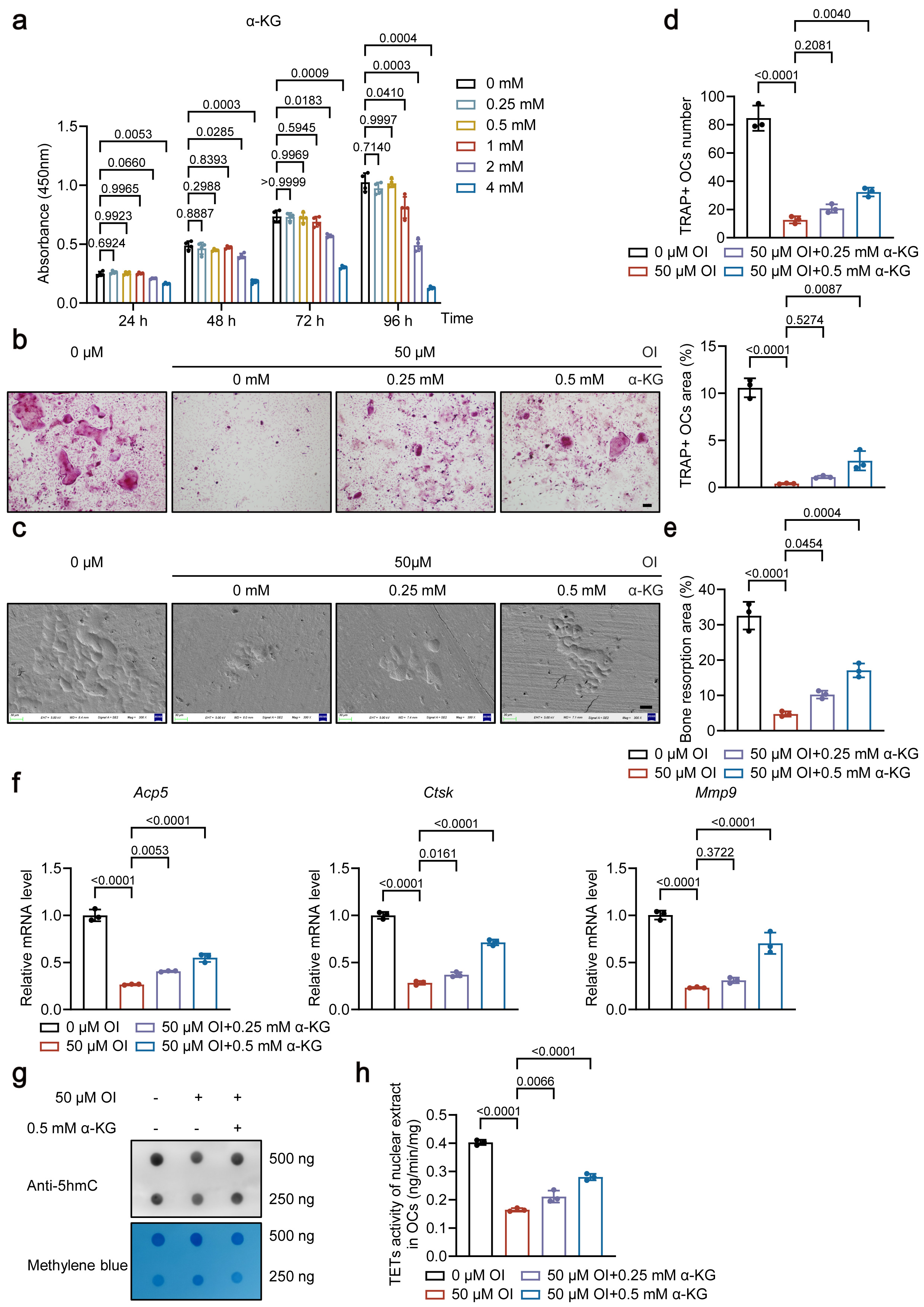
**Fig. S10 α-KG rescues the inhibitory effect of OI on osteoclasts.** **a** CCK-8 assay of BMMs cell viability at 0, 24, 48, 72, and 96 hours exposed to different concentrations of α-KG (n=4). **b**, **c** Representative images of TRAP staining (**b**) and pit formation assay (**c**) of OI-treated osteoclasts exposed to different α-KG concentrations (n=3). Scale bar: 5 μm (Top), 30 μm (Bottom). **d**, **e** Quantification of TRAP^+^ osteoclasts (**d**) at day 5 and quantification of bone resorption area (**e**) at day 7 from different treatments. (n=3). **f** Expression of *Acp5, Ctsk, Mmp9* mRNA in OI-treated BMMs induced for 5 days under different α-KG concentrations (n=3). **g** Dna dot blot assay of 5hmC levels in OI-treated BMMs added with α-KG during differentiation on day 3. **h** Assessment of TETs activity of nuclear extracts from OI-treated BMMs induced for osteoclast differentiation under various treatments for 3 days (n=3). Significant differences were determined by two-way ANOVA (**a**) and one-way ANOVA (**d**, **e**, **f**, **h**). Data represent means ± SD for each group. **a**-**h** n=3 biological independent experiments.


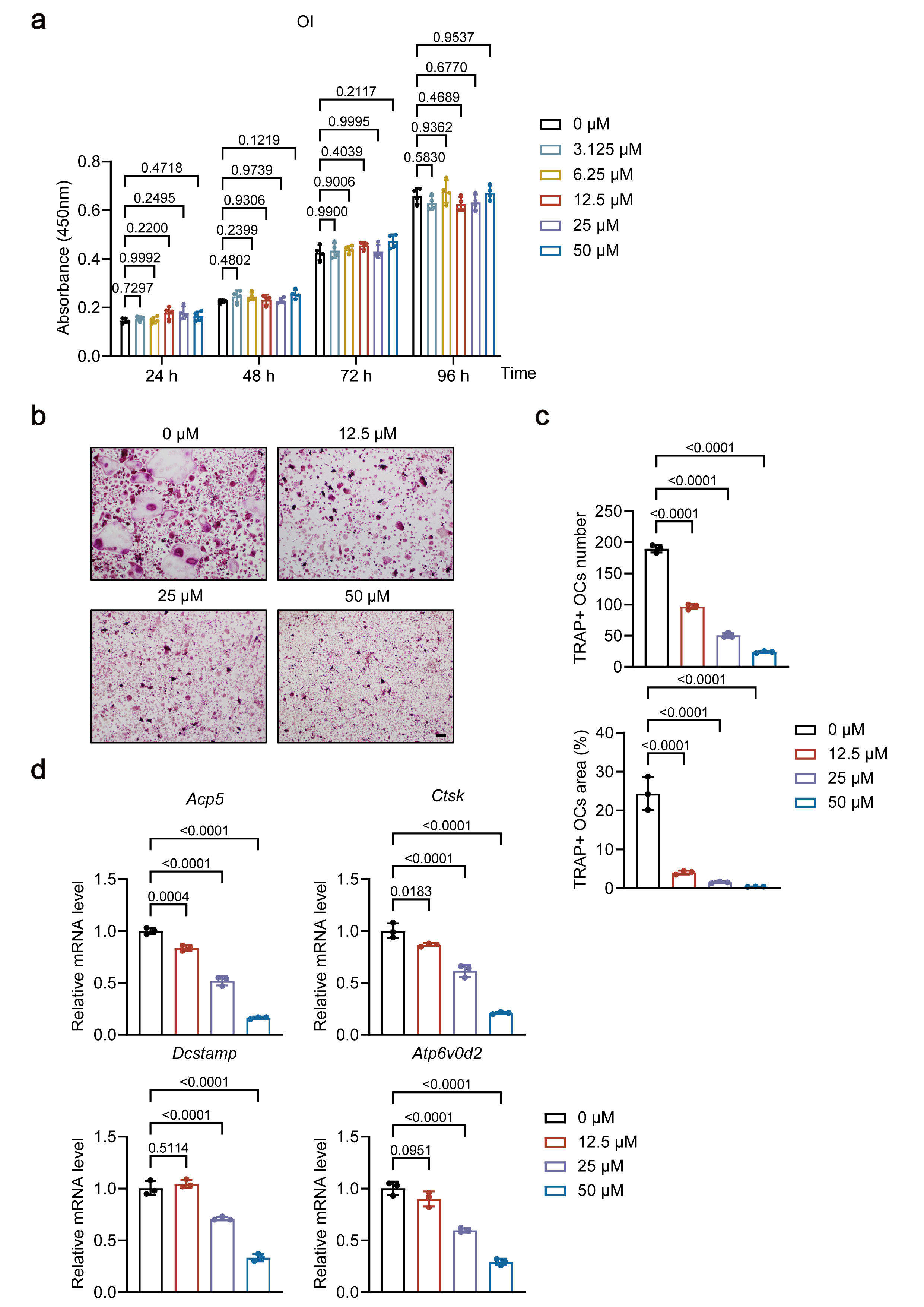
**Fig. S11 Exogenous OI inhibits osteoclast differentiation. a** CCK-8 assay of BMMs cell viability at 0, 24, 48, 72, and 96 hours exposed to different concentrations of OI (n=4). **b**, **c** Representative TRAP staining images (**b**) and quantification of TRAP^+^ osteoclasts (**c**) of mature osteoclasts at day 5 from different treatments (n=3). Scale bar: 5 μm. **d** Expression of osteoclast-associated genes at day 5 under treatment with 0, 12.5, 25, or 50 μM OI (n=3). Significant differences were determined by two-way ANOVA (**a**) and one-way ANOVA (**c**, **d**). Data represent means ± SD for each group. **a**-**d** n=3 biological independent experiments.


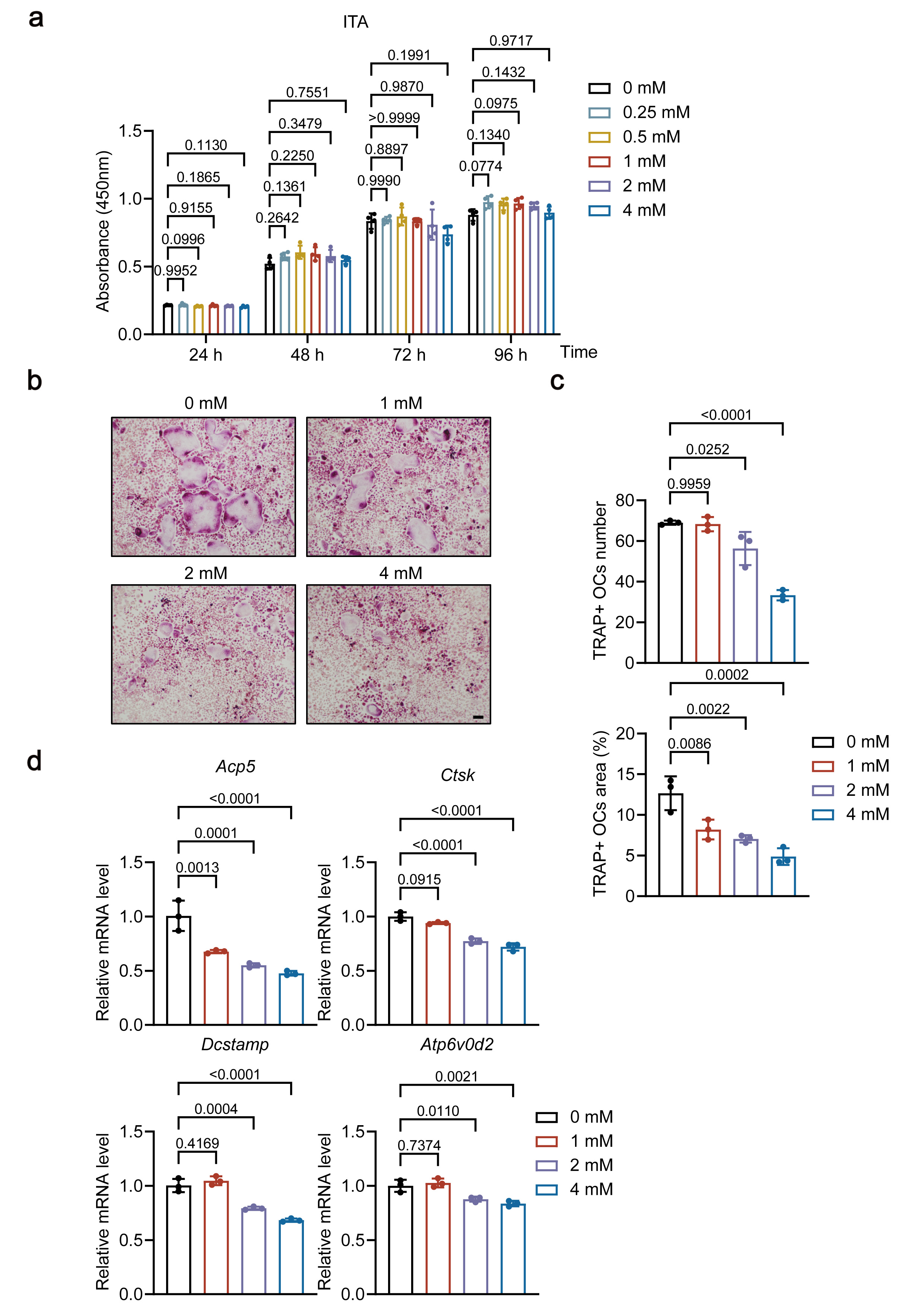
**Fig. S12 Exogenous ITA inhibits osteoclast differentiation. a** CCK-8 assay of BMMs cell viability at 0, 24, 48, 72, and 96 hours exposed to different concentrations of ITA (n=4). **b**, **c** Representative TRAP staining images (**b**) and quantification of TRAP^+^ osteoclasts (**c**) of mature osteoclasts at day 5 from different treatments (n=3). Scale bar: 5μm. **d** Expression of osteoclast-associated genes at day 5 under treatment with 0, 1, 2, or 4 mM ITA (n=3). Significant differences were determined by two-way ANOVA (**a**) and one-way ANOVA (**c**, **d**). Data represent means ± SD for each group. **a**-**d** n=3 biological independent experiments.


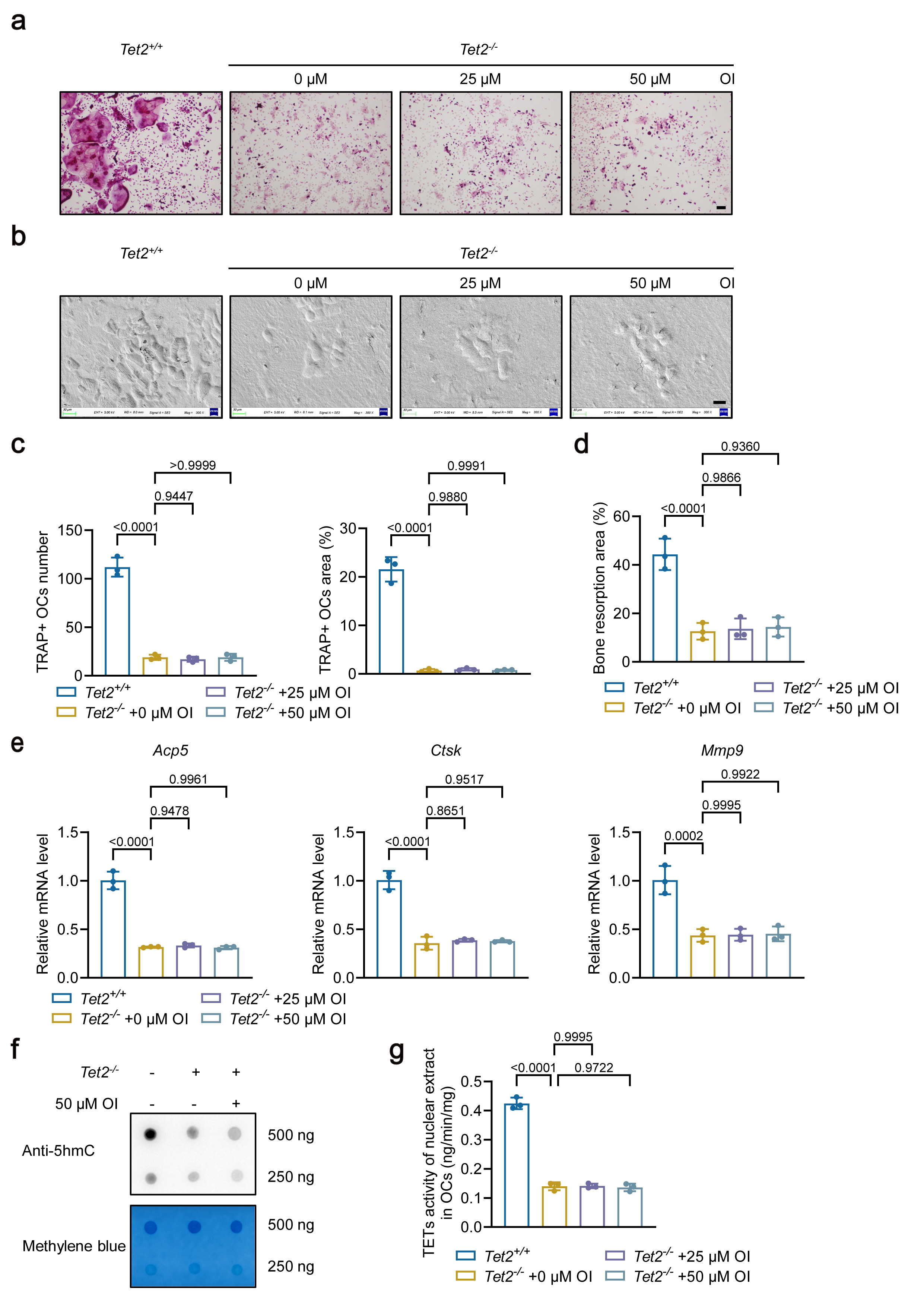
**Fig. S13 *Tet2^-/-^* BMMs show no response to the effects of OI during osteoclast differentiation. a**, **b** Representative images of TRAP staining (**a**) and pit formation assay (**b**) of mature osteoclasts induced from *Tet2^+/+^* and *Tet2^-/-^* BMMs with different concentrations of OI (n=3). Scale bar: 5 μm (Top), 30 μm (Bottom). **c**, **d** Quantification of TRAP^+^ osteoclasts (**c**) at day 5 and quantification of bone resorption area (**d**) at day 7 from different treatments (n=3). **e** Expression of *Acp5, Ctsk, Mmp9* mRNA in *Tet2^+/+^* and *Tet2^-/-^* BMMs induced for 5 days with different concentrations of OI (n=3). **f** Dna dot blot assay of 5hmC levels in *Tet2^+/+^* and *Tet2^-/-^* BMMs treated with 50 μM OI during differentiation on day 3 (n=3). **g** Assessment of TETs activity of nuclear extracts from *Tet2^+/+^* and *Tet2^-/-^* BMMs induced for osteoclast differentiation under different α-KG concentrations for 3 days (n=3). Significant differences were determined by one-way ANOVA (**c**, **d**, **e**, **g**). Data represent means ± SD for each group. **a**-**g** n=3 biological independent experiments.


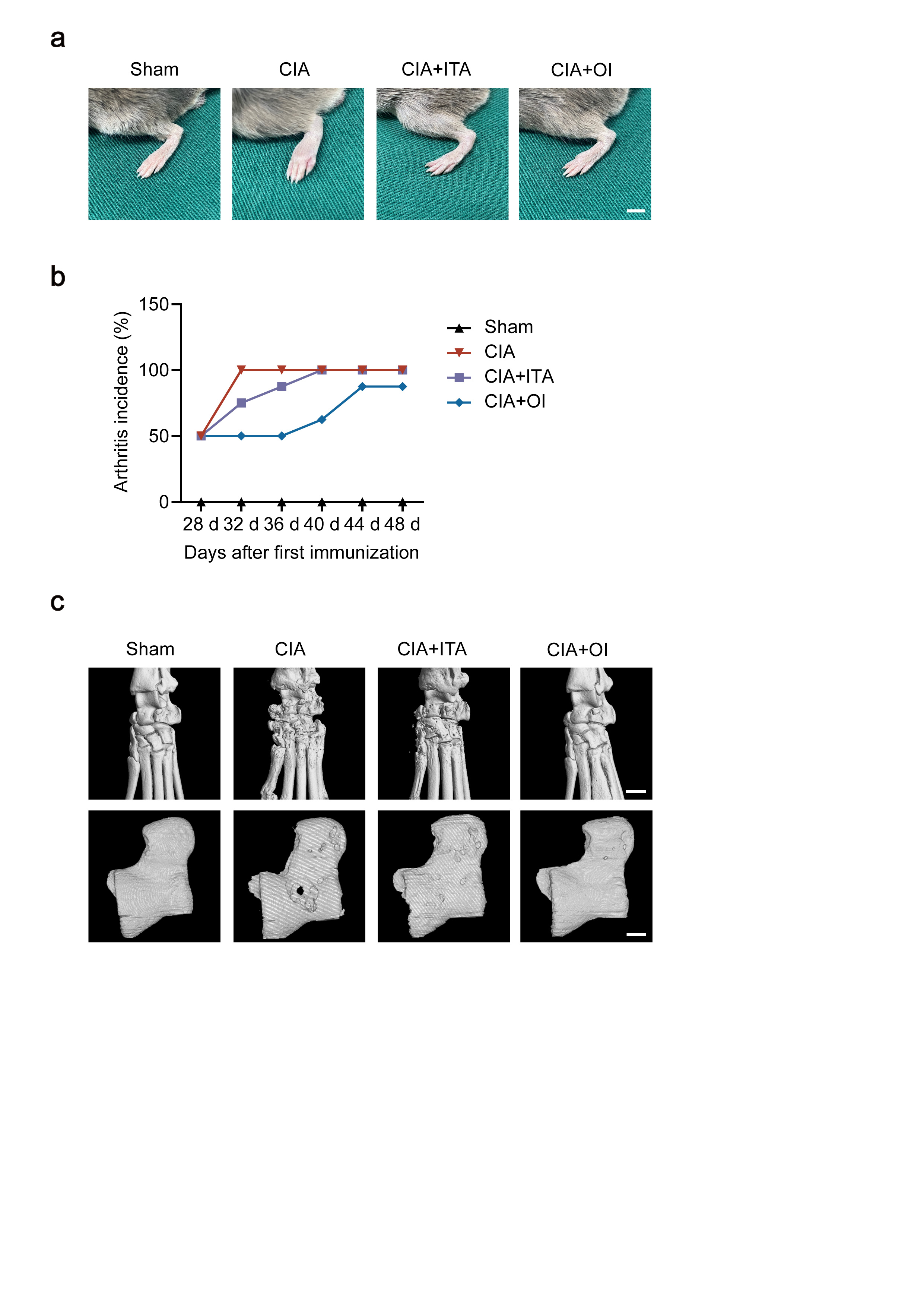


**Fig. S14 Assessment of arthritis severity and bone structure in CIA mice. a** Representative photographs of the CIA mice rear paws (n=8). Scale bar: 1 cm. **b** Arthritis incidence of CIA mice treated with cyclodextrin, ITA or OI (n=8). **c** Representative μCT reconstruction images of the rear paws and talus of Sham, CIA, CIA+ITA, CIA+OI mice (n=6). Scale bar: 1 mm (top), 100 μm (bottom).

**
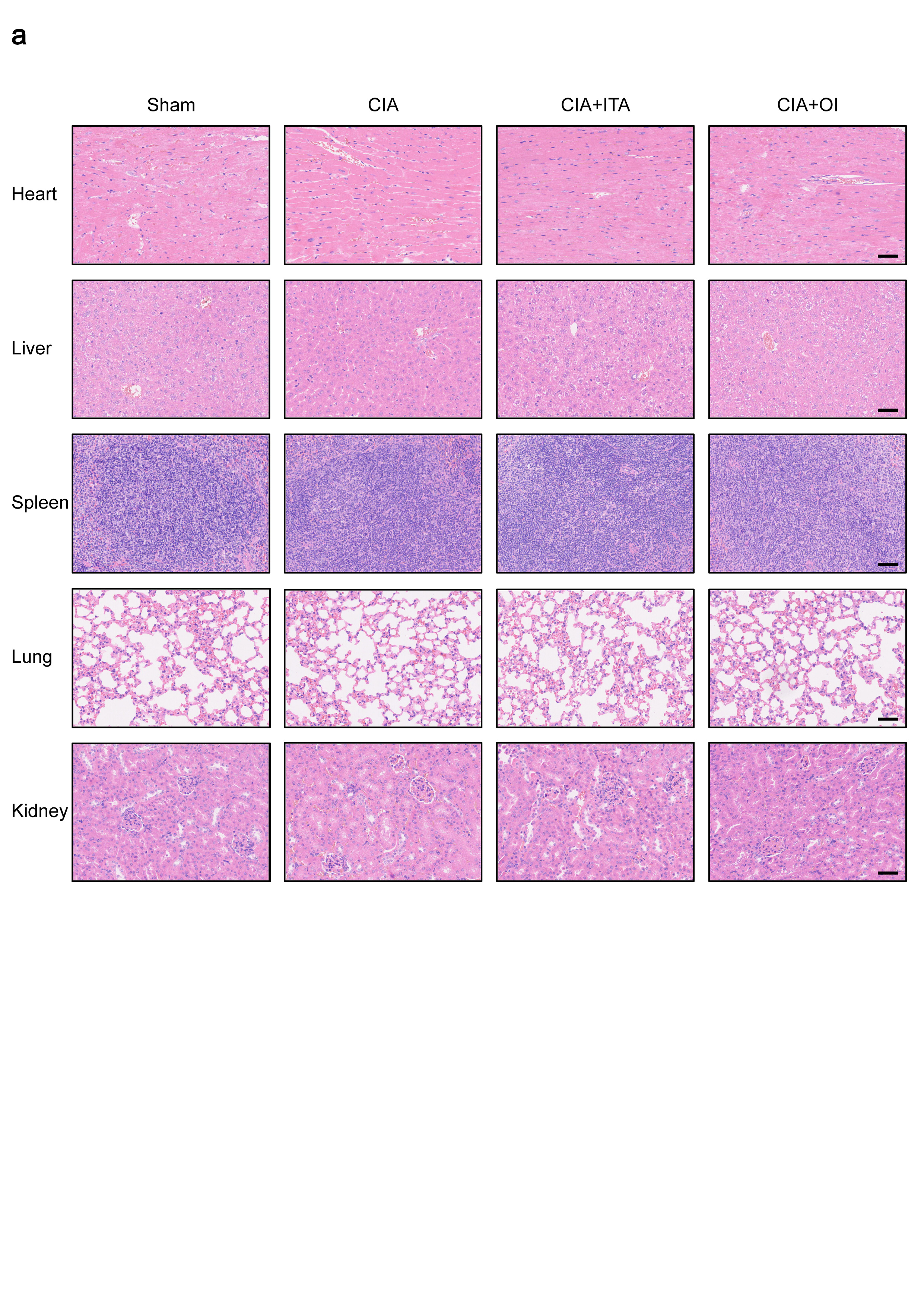
Fig. S15 ITA does not exhibit tissue toxicity in the heart, liver, spleen, lung, and kidney of CIA mice.** **a** Representative HE staining images of the heart, liver, spleen, lung, and kidneys from Sham, CIA, CIA+ITA, and CIA+OI mice (n=6). Scale bar: 50 μm.

**
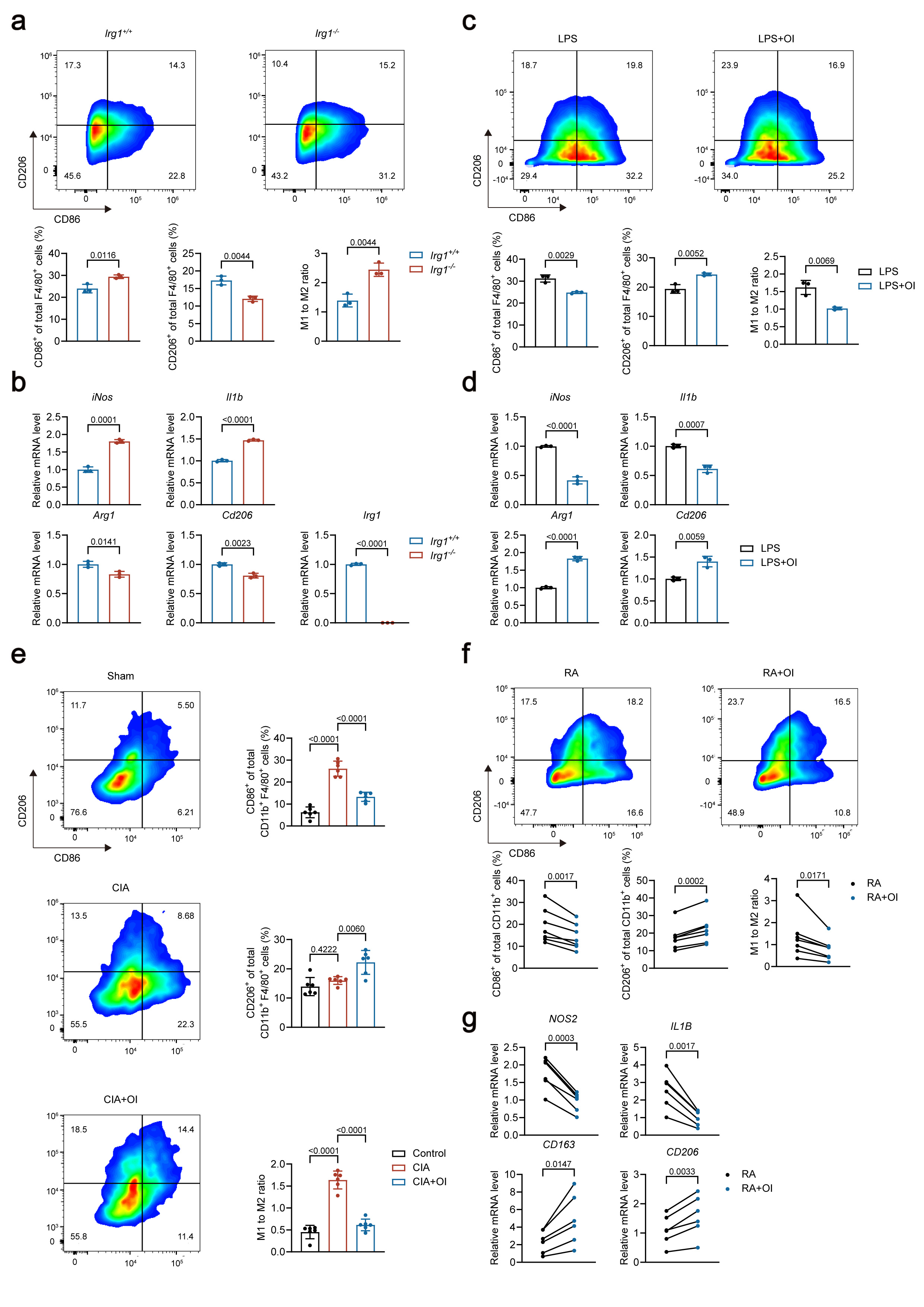
Fig. S16 ITA induces a more reparative phenotype in rheumatoid arthritis. a** Measurement of CD86 and CD206 expression in LPS-treated *Irg1^+/+^* and *Irg1^-/-^* BMMs (n=3). **b** Expression of *iNos*, *Il1b*, *Arg1*, *Cd206* and *Irg1* in LPS-treated *Irg1^+/+^* and *Irg1^-/-^* BMMs (n=3). **c** Measurement of CD86 and CD206 expression in LPS-treated BMMs with or without OI (n=3). **d** Expression of *iNos*, *Il1b*, *Arg1*, and *Cd206* in LPS-treated BMMs with or without OI (n=3). **e** Measurement of CD86 and CD206 expression in macrophages from the synovial compartment of CIA, CIA+OI mice (n=6). **f** Measurement of CD86 and CD206 expression in human RA-MDMs treated with or without OI (n=7). **g** Expression of *NOS2*, *IL1B*, *CD163* and *CD206* in human RA-MDMs treated with or without OI (n=6). Significant differences were determined by paired or unpaired Student’s t-test (**a**, **b**, **c**, **d**, **f**, **g**) and one-way ANOVA (**e**). Data represent means ± SD for each group. **a**-**d** n=3 biological independent experiments.


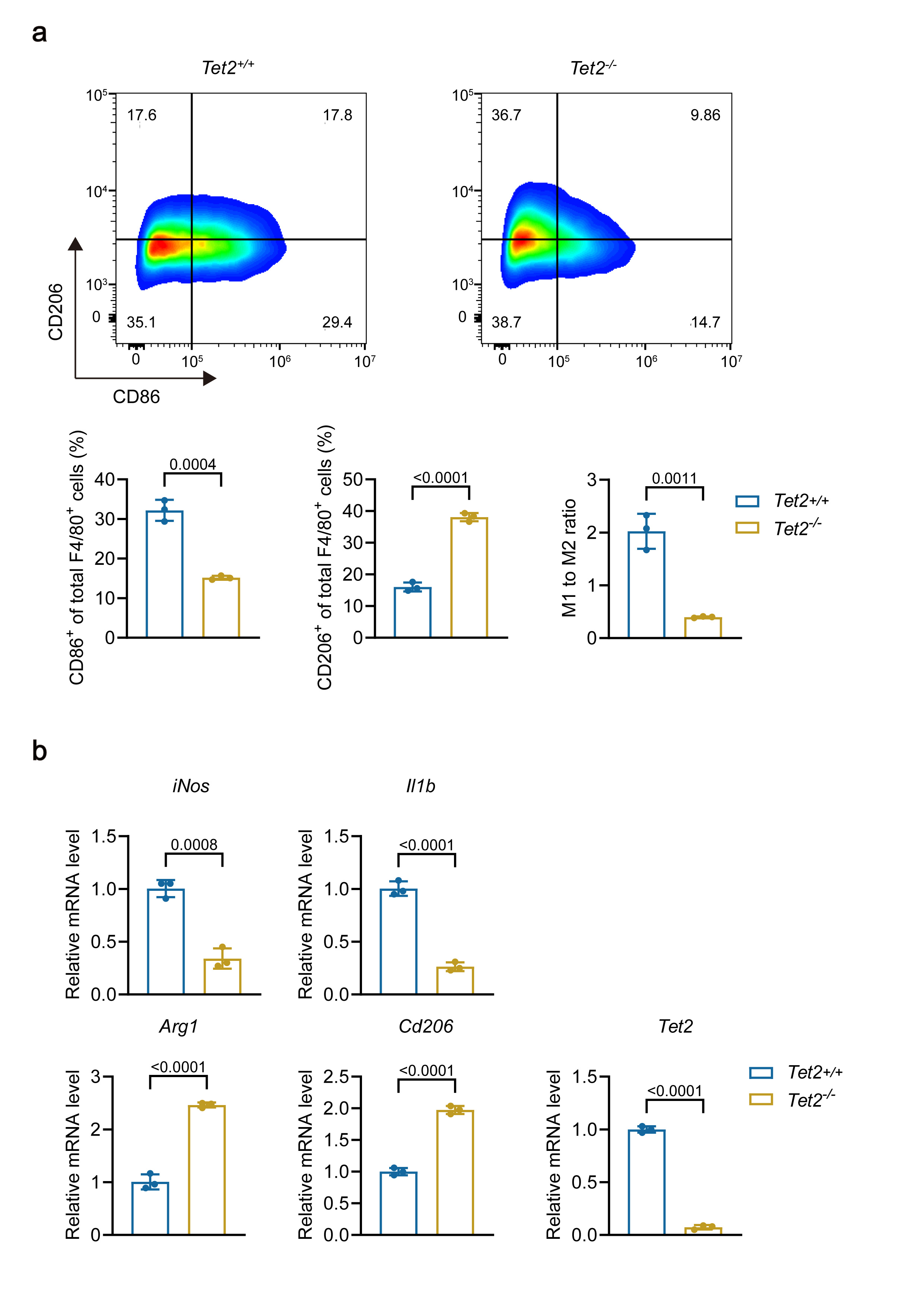
**Fig. S17 Deletion of Tet2 induces a more reparative phenotype. a** Measurement of CD86 and CD206 expression in LPS-treated *Tet2^+/+^* and *Tet2^-/-^* BMMs (n=3). **b** Expression of *iNos*, *Il1b*, *Arg1*, *Cd206* and *Tet2* in LPS-treated *Tet2^+/+^* and *Tet2^-/-^* BMMs (n=3). Significant differences were determined by Student’s t-test (**a**, **b**). Data represent means ± SD for each group. **a**, **b** n=3 biological independent experiments.
